# Designing complex Pb_3_SBr_*x*_I_4−*x*_ chalcohalides: tunable emission semiconductors through halide-mixing†

**DOI:** 10.1039/d3sc02733c

**Published:** 2023-10-16

**Authors:** Alison N. Roth, Yunhua Chen, Anuluxan Santhiran, Jemima Opare-Addo, Eunbyeol Gi, Emily A. Smith, Aaron J. Rossini, Javier Vela

**Affiliations:** a US DOE Ames National Laboratory Ames Iowa 50010 USA; b Department of Chemistry, Iowa State University Ames Iowa 50011 USA vela@iastate.edu

## Abstract

Chalcohalides are desirable semiconducting materials due to their enhanced light-absorbing efficiency and stability compared to lead halide perovskites. However, unlike perovskites, tuning the optical properties of chalcohalides by mixing different halide ions into their structure remains to be explored. Here, we present an effective strategy for halide-alloying Pb_3_SBr_*x*_I_4−*x*_ (1 ≤ *x* ≤ 3) using a solution-phase approach and study the effect of halide-mixing on structural and optical properties. We employ a combination of X-ray diffraction, electron microscopy, and solid-state NMR spectroscopy to probe the chemical structure of the chalcohalides and determine mixed-halide incorporation. The absorption onsets of the chalcohalides blue-shift to higher energies as bromide replaces iodide within the structure. The photoluminescence maxima of these materials mimics this trend at both the ensemble and single particle fluorescence levels, as observed by solution-phase and single particle fluorescence microscopy, respectively. These materials exhibit superior stability against moisture compared to traditional lead halide perovskites, and IR spectroscopy reveals that the chalcohalide surfaces are terminated by both amine and carboxylate ligands. Electronic structure calculations support the experimental band gap widening and volume reduction with increased bromide incorporation, and provide useful insight into the likely atomic coloring patterns of the different mixed-halide compositions. Ultimately, this study expands the range of tunability that is achievable with chalcohalides, which we anticipate will improve the suitability of these semiconducting materials for light absorbing and emission applications.

## Abstract 2

Quaternary chalcohalides, Pb_3_SBr_*x*_I_4−*x*_ (1 ≤ *x* ≤ 3), exhibit tunable photoluminescence emission through halide-mixing with variable amounts of Br^−^ and I^−^ anions in their structure.

## Graphical abstract

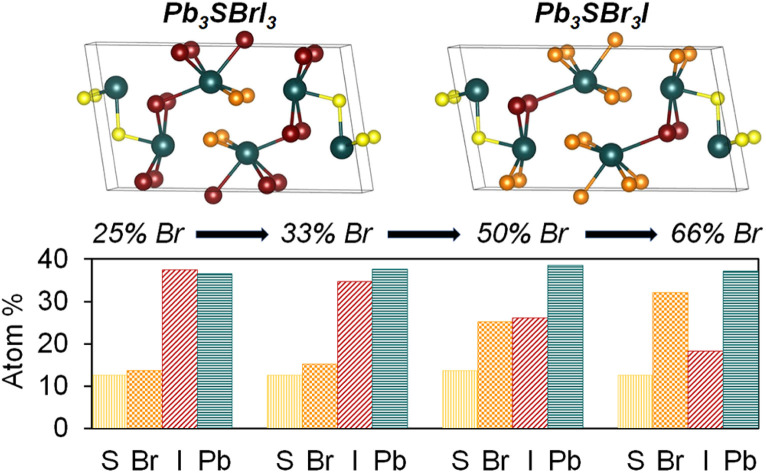

## Introduction

Lead halide perovskites are well known semiconducting materials for light harvesting and light emitting devices.^1^ Their impressive optical properties, such as tunable band gaps over the visible and near-IR regions^2^ and high photoluminescence quantum yields (PLQY)^3^ are often attainable due to their high defect tolerance.^4^ However, halide perovskites notoriously suffer from poor thermal and phase stability under irradiation due to the weak ionic interactions of metal–halide bonds, often resulting in halide migration, phase segregation, and PL instability.^5,6^ This challenge continues to limit full-scale implementation of halide perovskites into their anticipated technologically relevant applications.^7,8^

In light of this challenge, chalcohalides are gaining attention as promising alternative materials to halide perovskites.^9–11^ In principle, chalcohalides are expected to combine the optimal features of both chalcogenides and halide perovskites: that is, enhanced stability and light absorbing efficiency, respectively.^12,13^ Multinary chalcohalides have already demonstrated favorable structural and optical properties for photovoltaics.^14,15^ Other compositions are also being explored for applications including thermoelectrics,^16,17^ photocatalysis,^18^ and second harmonic generation.^19,20^ While solid-state reactions are primarily used to isolate these materials, the solution-phase chemistry of colloidal chalcohalides is rapidly expanding as a way to achieve better control over phase purity, particle size and morphology and, consequently, optoelectronic properties.^21–23^

**Fig. 1:**
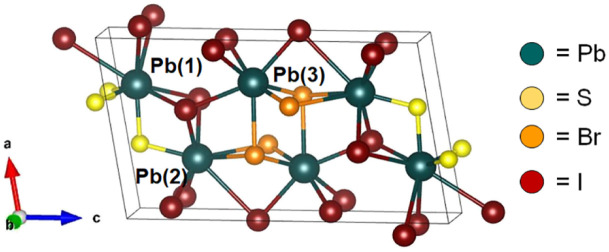
Unit cell of monoclinic (*P*2_1_/*m*) Pb_3_SBrI_3_.^25^

An exciting opportunity is to explore the effects of halide-alloying on chalcohalide semiconductors.^24^ Similar to mixed-halide perovskites (MHPs), introducing various types of halide ions into the structure of chalcohalides could lead to desirable band gap tunability while maintaining the enhanced stability and other characteristics displayed by chalcogenide-based materials. A suitable candidate for exploring this halide-alloying strategy is one of the few existing mixed-halide chalcohalides, *P*2_1_/*m* Pb_3_SBrI_3_. Recently isolated from a hydrothermal process, Pb_3_SBrI_3_ features a pseudo-two-dimensional layered structure with a narrow indirect band gap (Fig. 1).^25^ The low dimensionality of Pb_3_SBrI_3_ makes it unique among other mixed-halide chalcohalide compositions,^25,26^ as these features may be suggestive of anisotropic optical properties that are worth exploring in more detail.^27,28^ Furthermore, based on similarities with related quaternary chalcohalides, such as Pb_2_SbS_2_I_3_, we hypothesized that Pb_3_SBrI_3_ and other mixed-halide compositions may be accessible using a thiocyanate-based solution-phase synthesis.^21–23^

In this work, we explore the structural and optical properties of quaternary mixed-halide chalcohalides with the general composition Pb_3_SBr_*x*_I_4−*x*_ (1 ≤ *x* ≤ 3). X-ray diffraction (XRD) in combination with ^207^Pb solid-state (ss) NMR and electron microscopy (EM) confirm successful halide alloying and reveal that the materials adopt highly anisotropic morphologies. Interestingly, solution-phase spectroscopy and fluorescence microscopy show that the mixed-halide chalcohalides are photoluminescent both as an ensemble and at the single particle level. Their band gap and PL emission energies blue-shift as the content of bromine relative to iodine increases. We used DFT calculations to assess the atomic coloring pattern of the bromine-rich mixed-halide compositions and their corresponding optical properties. Our results suggest that halide-alloying may be a useful strategy for further modifying and enhancing the opto-electronic properties of chalcohalide semiconductors.

## Results and discussion

### Chalcohalide synthesis and halide mixing

**Scheme 1:**
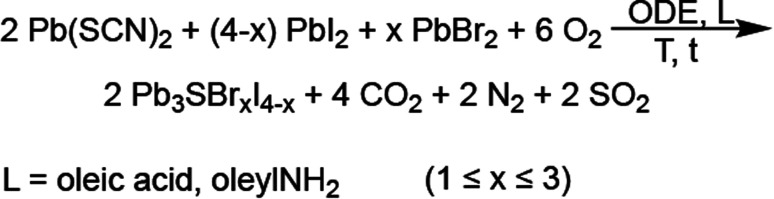
Solution-phase synthesis of quaternary mixed-halide chalcohalides.^23,29^

Pb_3_SBrI_3_ adopts a monoclinic (*P*2_1_/*m*) structure composed of three lead sites with possible coordination environments of [PbS_3_I_5_], [PbSBr_2_I_5_], and [PbBr_3_I_4_], respectfully (Fig. 1). We prepare Pb_3_SBrI_3_ from solution using a heat-up approach—additional Experimental details appear in the ESI File† (Scheme 1). Standard starting mixtures consist of 19 mM Pb(SCN)_2_, 19–57 mM each of PbBr_2_ and PbI_2_, 0.25 mL each of oleylamine (oleylNH_2_) and oleic acid, and 10 mL of 1-octadecene (ODE). Upon heating to temperatures above 150 °C, the reaction mixture initially undergoes a distinct color change from light yellow to dark red, indicating decomposition of the thiocyanate precursor.^29,30^ Once the temperature reaches 180 °C, the reaction mixture gradually transforms to a vivid yellow-orange color over the course of ≥1 h, indicating the formation of the chalcohalide. Varying the initial concentrations of PbI_2_ and PbBr_2_, respectively, from 57 mM and 19 mM, to 38 mM and 19 mM, 19 mM and 19 mM, and 19 mM and 38 mM enables the preparation of Pb_3_SBr_*x*_I_4−*x*_ (1 ≤ *x* ≤ 3) phases with nominal halide synthetic loadings of 25%, 33%, 50%, and 66% Br, respectively. These reactions generally occur at 180–200 °C for 60–90 min, resulting in bright yellow-colored suspensions (Table 1, see ESI†).

**Table 1:** Synthesis of mixed-halide chalcohalides

Precursors		Ligands^a^ (mL)	*T* (°C)	*t* (min)	Product^b^	Band gap^c^ (eV)	Width^d^ (Scherrer)^e^ (*w*, nm)	Length^d^ (*l*, μm)	Aspect ratio (*l*/*w*)
Pb–S (mmol)	PbX̲_2_ (mmol)								
Pb(SCN)_2_ (0.2)	Br (0.2), I (0.6)	OleylNH_2_ (0.25)	180	90	Pb_2.9_SBr_1.1_I_3.0_	2.22	94 ± 22 (70 ± 31)	1.0 ± 0.4	11
		Oleic acid (0.25)							
Pb(SCN)_2_ (0.2)	Br (0.2), I (0.4)	OleylNH_2_ (0.25)	180	60	Pb_3.0_SBr_1.2_I_2.8_	2.26	150 ± 30 (129 ± 56)	3.1 ± 1.3	21
		Oleic acid (0.25)							
Pb(SCN)_2_ (0.2)	Br (0.2), I (0.2)	OleylNH_2_ (0.25)	180	60	Pb_2.8_SBr_1.8_I_1.9_	2.29	170 ± 28 (152 ± 92)	3.0 ± 0.9	18
		Oleic acid (0.25)							
Pb(SCN)_2_ (0.2)	Br (0.4), I (0.2)	OleylNH_2_ (0.25)	200	60	Pb_3.0_SBr_2.6_I_1.5_	2.33	270 ± 80 (245 ± 116)	4.3 ± 1.5	16
		Oleic acid (0.25)							

a ODE = 1-octadecene (10 mL); oleylNH_2_ = oleylamine.

b From EDS.

c From Tauc plots.^50^

d From SEM.

e From XRD.

**Fig. 2:**
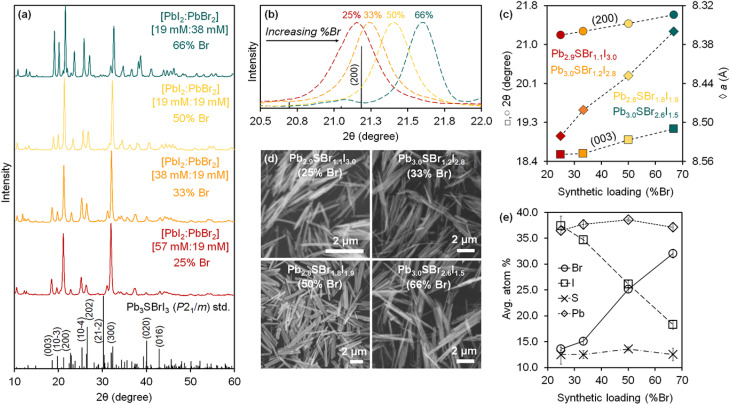
(a) Powder XRD patterns of mixed-halide chalcohalides prepared with different concentrations of PbI_2_ and PbBr_2_ (see details in ESI†). (b) Normalized XRD patterns showing the shift in the (200) reflection to higher 2*θ* with increasing %Br. (c) Position of the (200) and (003) reflections and *a* lattice parameter as a function of %Br. (d) SEM images showing the rod-like morphology of the mixed-halide chalcohalides: Pb_2.9_SBr_1.1_I_3.0_, Pb_3.0_SBr_1.2_I_2.8_, Pb_2.8_SBr_1.8_I_1.9_, and Pb_3.0_SBr_2.6_I_1.5_. (e) EDS analysis of mixed-halide chalcohalides as a function of %Br.

Powder X-ray diffraction (XRD) shows that the reaction with a synthetic loading of 25% Br—relative to total halides—results in the known *P*2_1_/*m* Pb_3_SBrI_3_ phase, although a few reflections appear broadened and/or selectively attenuated (Fig. 2). We hypothesized that this may be caused by size (Scherrer) effects as well as by anisotropic (preferred) particle growth, similar to what is observed for mixed-cation chalcohalide nanorods^23,31^ and Pb_5_S_2_I_6_ nanoparticles.^32^ Indeed, scanning electron microscopy (SEM) confirms that the mixed-halide chalcohalides consist of elongated, high-aspect ratio, rod- and needle-like particles (Fig. 2d). The average rod length of the mixed-halides is between 1–4 μm, and the average rod diameter 100–300 nm, in close agreement with their average Scherrer size from XRD (see Table 1). In general, the average rod diameter increases with higher %Br synthetic loading, suggesting a higher reactivity for the bromide precursor.^33^

Powder X-ray diffraction (XRD) shows that higher Br synthetic loadings between 33% and 66% result in crystalline solids with patterns similar to that of Pb_3_SBrI_3_. However, the diffraction peaks in these patterns progressively shift to higher 2*θ* values—corresponding to lower *d*-spacings—with the largest shift observed for the material with the highest (66%) Br loading (Fig. 2a–c). These XRD features are very characteristic of similar materials such as mixed-halide perovskites (MHPs), where XRD reflections shift to wider (2*θ*) angles with increasing incorporation of smaller halide ions.^34–36^ The shift in XRD reflections indicates a steady reduction of lattice parameters and, consequently, a compression of the unit cell volume relative to the original Pb_3_SBrI_3_ crystal structure. Thus, the volume compression observed with increasing %Br is consistent with smaller Br^−^ (*r*_crys_ = 1.82 Å) replacing larger I^−^ (*r*_crys_ = 2.06 Å)^37^ within the structure, supporting the idea of successful halide-alloying in Pb_3_SBr_*x*_I_4−*x*_ (1 ≤ *x* ≤ 3). Interestingly, attempts to incorporate more Br into the chalcohalide were unsuccessful. For example, 75% or higher Br synthetic loadings lead to powder XRD patterns that showed crystalline impurities along with the quaternary material.

Energy dispersive spectroscopy (EDS) confirms mixed-halide phases within the successful alloying regime become richer in Br and poorer in I with increasing Br loading, yielding quaternary phases corresponding to Pb_2.9_SBr_1.1_I_3.0_ (25% Br), Pb_3.0_SBr_1.2_I_2.8_ (33% Br), Pb_2.8_SBr_1.8_I_1.9_ (50% Br), and Pb_3.0_SBr_2.6_I_1.5_ (66% Br), respectively (Fig. 2e). High-resolution transmission electron microscopy (HRTEM) of Pb_2.8_SBr_1.8_I_1.9_ shows that (002) and (001) lattice planes stack along the narrow diameter dimension of the rods while (11̄1) lattice planes stack along the longer rod length (see ESI†). These findings are consistent with the rods growing along the [010] *b*-direction (rod length) and their diameter being along the [001] *c*-direction (rod width). In a few cases, high resolution TEM revealed a few small ≤4–5 nm spots—PbI_2_ or PbS—along the rods. Nonetheless, EM confirms that the quaternary chalcohalides are indeed alloyed with bromine and iodine, in agreement with the XRD results.

### Effect of precursor on phase evolution

**Fig. 3:**
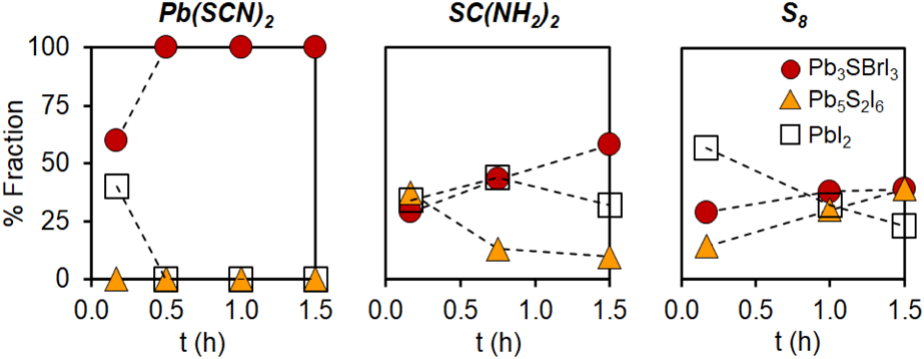
Phase evolution of mixed-halide quaternary chalcohalide Pb_3_SBrI_3_*vs.* ternary and binary impurities over time as a function of sulfur precursor used (under identical conditions to those in the first row of Table 1; see Experimental in the ESI†).

As part of our initial efforts aimed at finding suitable syntheses for mixed chalcohalide semiconductors, we screened different sulfur precursors. Under identical conditions to those in the first row of Table 1 (see ESI†), the use of Pb(SCN)_2_ results in quick formation of chalcohalide; only the desired Pb_3_SBrI_3_ quaternary phase is observed after 0.5 h reaction (Fig. 3). In contrast, both thiourea (SC(NH_2_)_2_) and elemental sulfur (S_8_) react much more slowly, achieving only *ca.* 40–50% quaternary phase purity after 1.5 h reaction. In both cases, unreacted crystalline PbI_2_ is still present; further, the ternary chalcohalide Pb_5_S_2_I_6_ is also observed. Because the appearance of Pb_3_SBrI_3_ coincides with the disappearance of Pb_5_S_2_I_6_, we speculate that the formation of the lower order, ternary phase may precede the formation of the desired, mixed-halide, quaternary phase.

We believe the key to understanding these differences is the existence of preformed Pb–S bonds in Pb(SCN)_2_.^21,23,29^ According to the literature, Pb–S bonds (Δ*H*_diss_ = 398 kJ mol^−1^) are much stronger than Pb–X bonds (X = Br or I, Δ*H*_diss_ = 248–194 kJ mol^−1^).^38^ The stronger, more robust Pb–S bonds thus require a higher activation energy to make (or break) than the Pb-halide bonds (this trend is also evident when the solubility product constants of the corresponding binaries are used as surrogates for a stability measure: *K*_sp_ = 3.0 × 10^−28^ for PbS, 9.8 × 10^−9^ for PbI_2_, and 6.6 × 10^−6^ for PbBr_2_).^38^ Because Pb(SCN)_2_ already contains preformed Pb–S bonds, it is able to more quickly react and deliver [Pb–S] building blocks needed for the effective nucleation and growth of multinary chalcohalide nanocrystals. Thiourea and elemental sulfur lack this advantage, and must overcome a larger kinetic barrier to form Pb–S bonds; therefore, these alternative precursors are inferior in kinetics, phase purity, and yield compared to Pb(SCN)_2_.

### Enhanced stability of chalcohalides

**Fig. 4:**
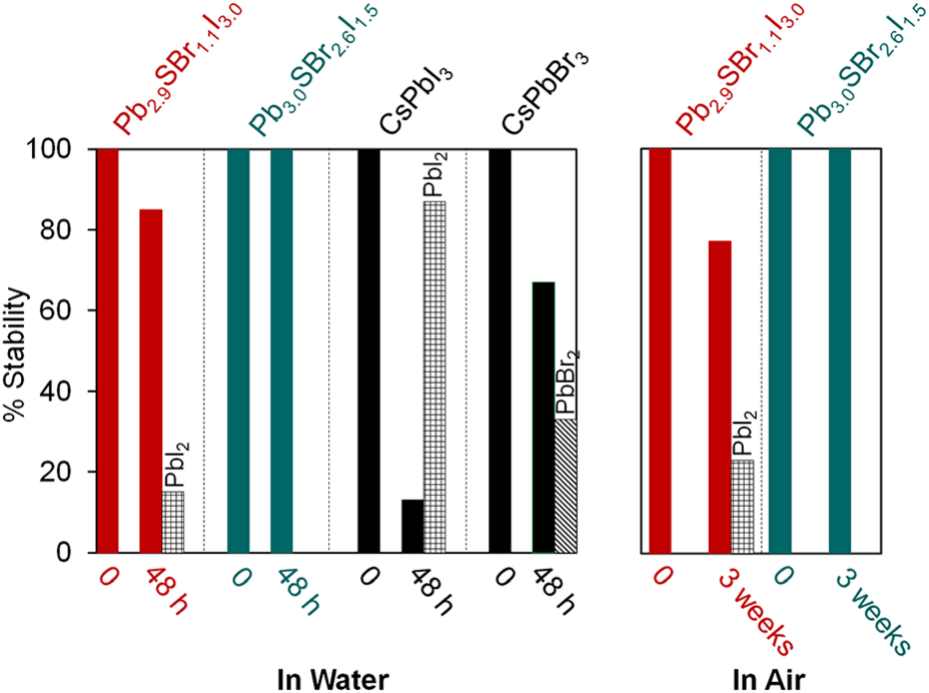
Relative stability of Pb_2.9_SBr_1.1_I_3.0_ and Pb_3.0_SBr_2.6_I_1.5_ over time in water (left) and under air (right).

In order to further assess the stability of mixed-halide chalcohalides compared to traditional halide perovskites, we compared them to standard samples of CsPbBr_3_ and CsPbI_3_ as reference materials.^35^ After stirring all individual samples in water for 48 h, the chalcohalides demonstrate impressive stability compared to the halide perovskites. Specifically, powder XRD reveals that Pb_2.9_SBr_1.1_I_3.0_ and Pb_3.0_SBr_2.6_I_1.5_ remain largely intact with ≥85% of the original semiconductor remaining (Fig. 4, see ESI†) after water treatment; in contrast, CsPbI_3_ and CsPbBr_3_ easily decompose to PbI_2_ and PbBr_2_, respectively. Furthermore, the chalcohalides display good stability over time under standard atmospheric conditions. After 3 weeks under air at room temperature (21 °C), Pb_3.0_SBr_2.6_I_1.5_ remains intact without any evidence of crystalline impurities or decomposition, while Pb_2.9_SBr_1.1_I_3.0_ only slightly decomposes to PbI_2_ over time. Thus, the more bromine-rich compositions appear to have higher overall stability compared to the more iodine-rich compositions, suggesting that stability increases with the incorporation of the slightly harder, more electronegative bromine ions into the structure. These findings mirror similar trends in the photo- and thermal stability along the CsPbX_3_ series (X = Cl, Br, or I).^39,40^ From these analyses, we conclude that mixed-halide chalcohalides possess better ambient and moisture stability compared to halide perovskites, which we anticipate will be help stimulate their incorporation into energy conversion devices.

### Surface carboxylates, halides, and hydrogen bonded amines

**Fig. 5:**
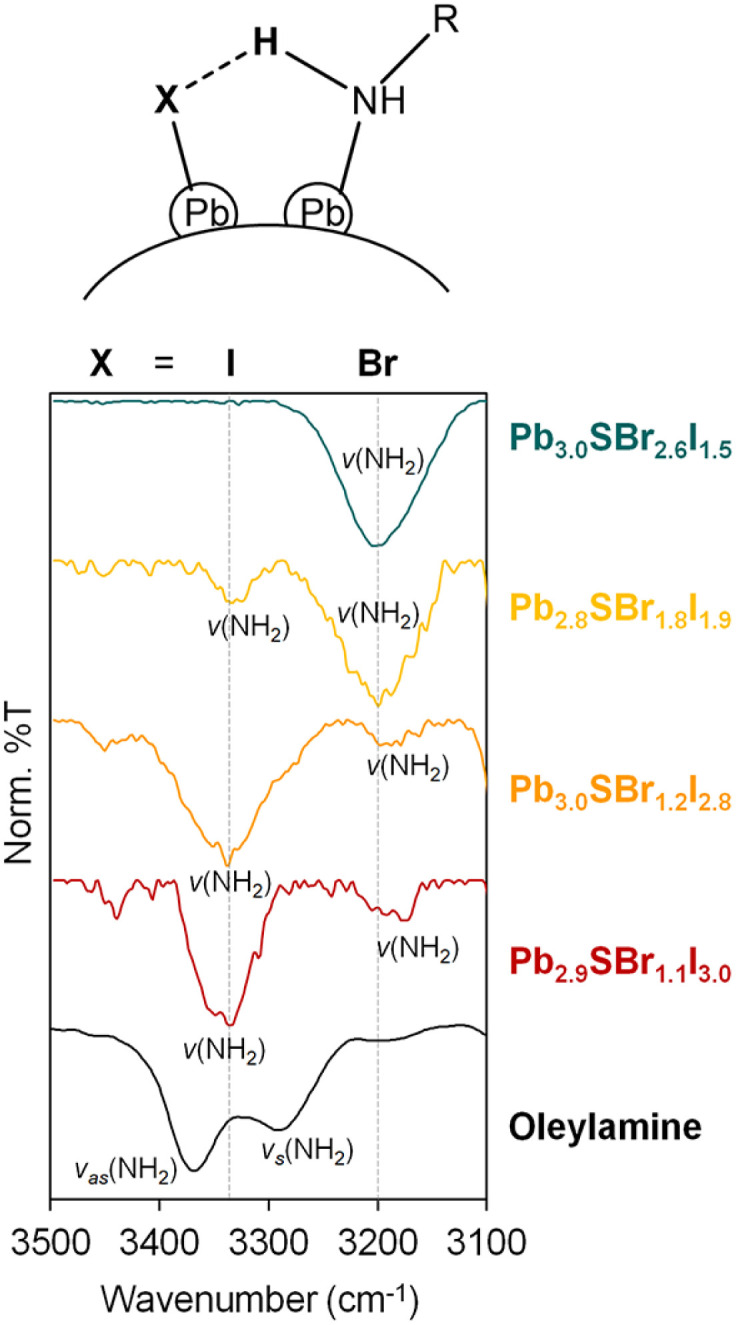
IR spectra of mixed-halide chalcohalides compared to oleylamine in the 3100–3500 cm^−1^ region.

To determine if any of the ligands used during synthesis coordinates to the chalcohalide particles after purification, we studied their surface chemistry using infrared (IR) spectroscopy (Fig. 5, see ESI†). A combination of *v*(N–H) (3400–3200 cm^−1^) and *ν*(C–N) (1100 cm^−1^) vibrations indicates the presence of surface-bound amine ligands, which are commonly present on chalcogenide nanocrystals,^41,42^ as well as halides (see below). Interestingly, the *v*(N–H) stretch at 3330 cm^−1^ in Pb_2.9_SBr_1.1_I_3.0_ gradually weakens as additional Br is incorporated into the lattice. This is accompanied by the appearance of a new *v*(N–H) stretch at ∼3200 cm^−1^, which gradually increases with Br incorporation and becomes the dominant peak in the most bromine-rich composition, Pb_3.0_SBr_2.6_I_1.5_.

Free carboxylic acid fails to account for any of these peaks, as the characteristic C=O band at ∼1700 cm^−1^ indicative of a protonated carboxylate (–COOH) is absent from all samples. Rather, we hypothesize that the two *v*(N–H) peaks correspond to two distinct surface-bound primary amines, each of which is hydrogen bonded to a surface-bound halide. This can be primarily iodide as in Pb_2.9_SBr_1.1_I_3.0_, or bromide as in Pb_3.0_SBr_2.6_I_1.5_, as shown at the top of Fig. 5. Such red-shift (to lower energy) of a hydrogen bonded amine 
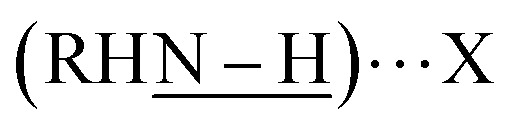
 when X goes from I (3330 cm^−1^) to the more electronegative Br (3200 cm^−1^) has ample precedent in the literature, for example in methylammonium halides as well as in multiple organometallic compounds.^43–47^ In addition to halides and hydrogen-bonded amine ligands, we also observe vibrations between 1400–1600 cm^−1^ that correspond to *v*_as_(COO–) and *v*_s_(COO–). The small separation between these two bands of less than 100 cm^−1^ indicates that chelating (*η*^2^) carboxylate (oleate) ligands are also bound to the particle surface.^48,49^

### Ensemble and single particle photoluminescence

**Fig. 6:**
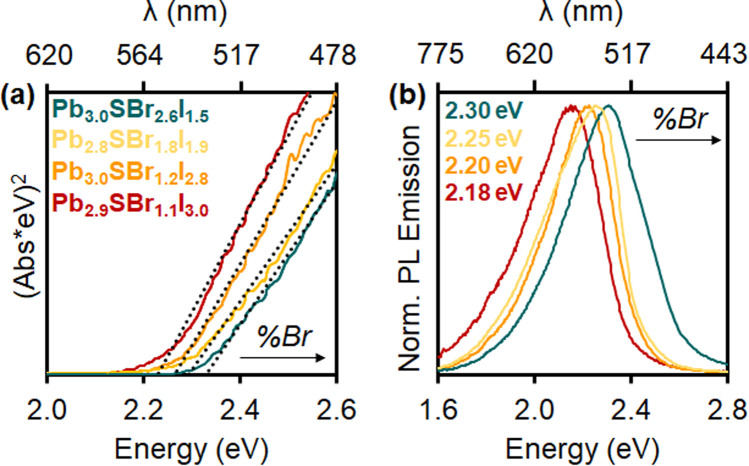
(a) Tauc plots and (b) photoluminescence spectra of Pb_2.9_SBr_1.1_I_3.0_ (red), Pb_3.0_SBr_1.2_I_2.8_ (orange), Pb_2.8_SBr_1.8_I_1.9_ (yellow), and Pb_3.0_SBr_2.6_I_1.5_ (green).

Diffuse reflectance demonstrates that the chalcohalides absorb strongly within the visible region, consistent with their bright yellow color (Fig. 6). Indirect Tauc plots^50^—assuming the mixed-halides to be indirect semiconductors based on the electronic structure of *P*2_1_/*m* Pb_3_SBrI_3_—show that the band gaps gradually widen as more Br is incorporated into the structure. Specifically, the band gap changes from 2.22 eV (Pb_2.9_SBr_1.1_I_3.0_) to 2.26 eV (Pb_3.0_SBr_1.2_I_2.8_), 2.29 eV (Pb_2.8_SBr_1.8_I_1.9_), and 2.33 eV (Pb_3.0_SBr_2.6_I_1.5_), respectively (Table 1). These results are also consistent with the increased band gap energy observed in mixed-halide perovskites (MHPs) with the incorporation of more electronegative—and smaller—halide ions.^34,35,51^

Photoluminescence (PL) measurements reveal that the mixed-halide chalcohalides are emissive in the visible region, with PL emission maxima (PL_max_) between 540 and 570 nm (Fig. 6). Furthermore, PL_max_ values gradually blue-shift with increasing Br-loading and content, in close agreement with the respective band gap energies. Critically, in addition to solution-phase (ensemble) PL measurements, we also successfully measured PL of single mixed-halide chalcohalide particles using fluorescence microscopy. Steady shape-correlated PL emission lacking blinking or OFF periods is observed over time under continuous photoexcitation (see ESI†).^23^ This finding strongly suggests that the PL originates from the rods themselves rather than from lower order impurities or unreacted precursor(s).

### ^207^Pb ssNMR spectroscopy

**Fig. 7:**
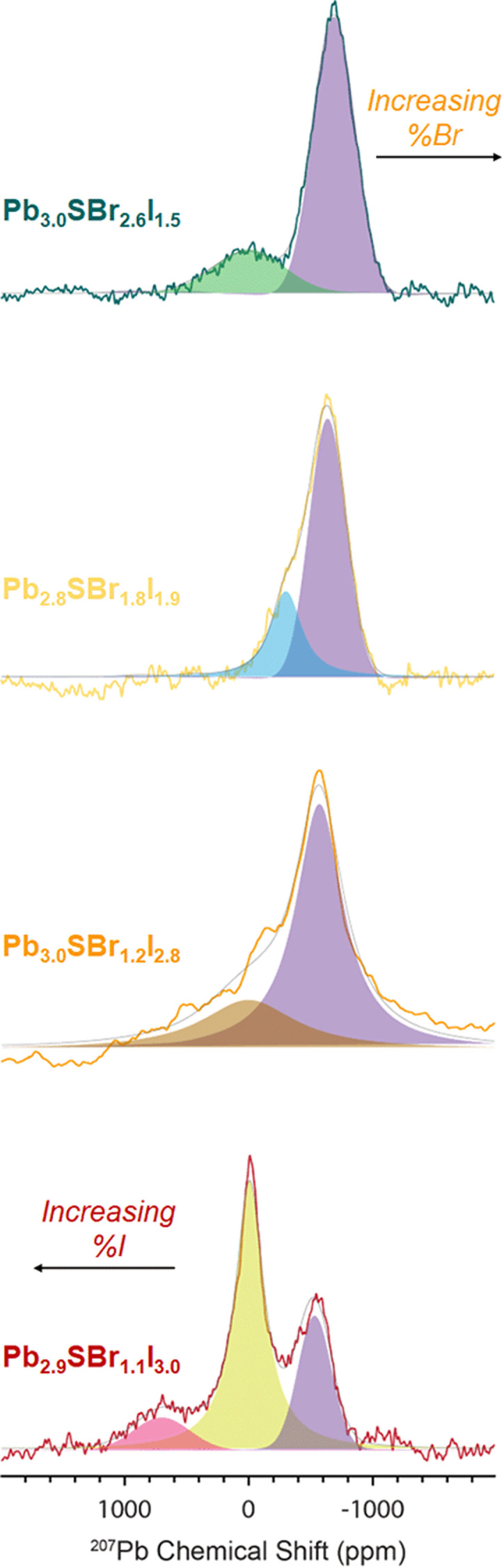
^207^Pb spin echo ssNMR spectra of Pb_2.9_SBr_1.1_I_3.0_, Pb_3.0_SBr_1.2_I_2.8_, Pb_2.8_SBr_1.8_I_1.9_, and Pb_3.0_SBr_2.6_I_1.5_.

To probe the chemical speciation of the three mixed-halide chalcohalides, we resorted to solid-state ^207^Pb NMR spectroscopy (Fig. 7).^34,52–54^ Our data show that the NMR signals generally shift to more negative chemical shifts with the incorporation of more electronegative Br^−^ ions into the structure, which is consistent with what is observed in binary lead halides,^54,55^ as well as in mixed-halide perovskites (MHPs).^56^ Specifically, three observable ^207^Pb NMR signals for Pb_2.9_SBr_1.1_I_3.0_ resonate at *ca.* 701.6, −7.9, and −531.7 ppm respectively, with an integral ratio of ∼1 : 6.5 : 2.6 (note: the ^207^Pb NMR spectra are not quantitative because we cannot measure the ^207^Pb longitudinal (*T*_1_) and transverse relaxation time constants 
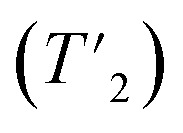
 due to low sensitivity). We assign the three distinct signals to the fully occupied sites Pb(1), Pb(2), and Pb(3) (see ESI†). While one could expect other compositions to also show three peaks based on the Pb environments in the chalcohalide crystal structure, the specific chemical shifts of some of these environments are closely positioned, appearing as a single peak. We are able to resolve two distinct Pb chemical shifts in the more bromine-rich compositions. These broadened signals appear at *ca.* −302.0 and −639.6 ppm with a 1 : 2.5 integral ratio in Pb_2.8_SBr_1.8_I_1.9_ and at −3.02 and −691.1 ppm with a 1 : 3.7 integral ratio in Pb_3.0_SBr_2.6_I_1.5_. We conclude that the chalcohalide samples are free of any PbBr_2_ impurity because of the absence of a peak at *ca.* −980 ppm.^55,57^ Further, while it is difficult to discern the presence of PbI_2_ based on ssNMR chemical shifts alone,^58^ the XRD patterns of all of the chalcohalides are inconsistent with the presence of any such crystalline binary impurities.

### Atom coloring and electronic calculations

**Fig. 8:**
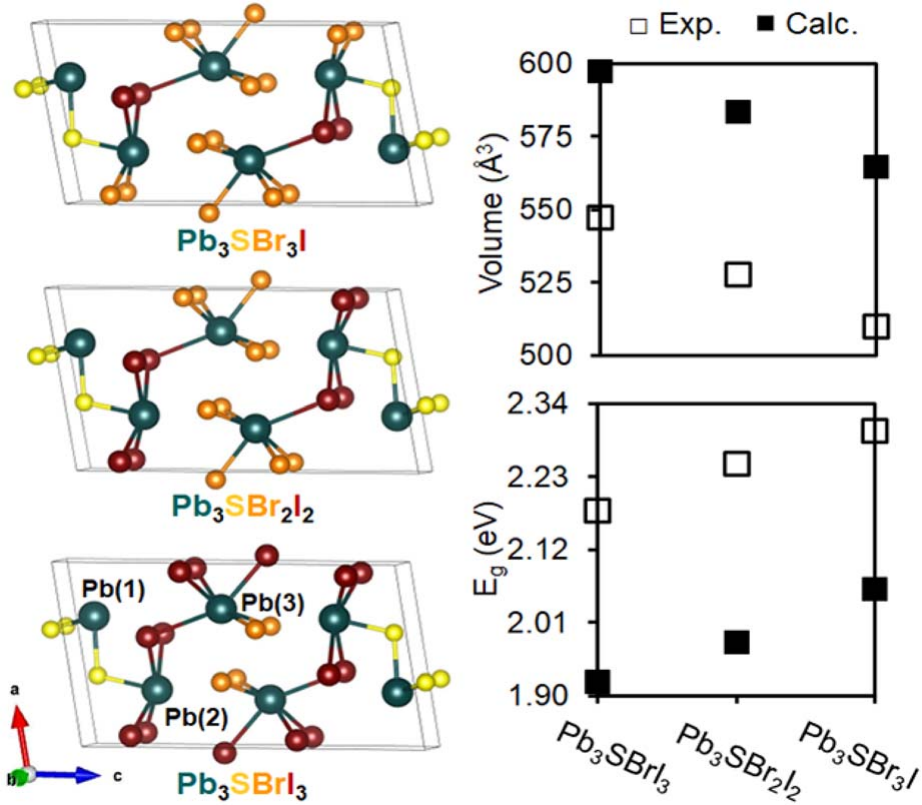
Atomic coloring patterns of the lowest energy unit cells of Pb_3_SBrI_3_, Pb_3_SBr_2_I_2_, and Pb_3_SBr_3_I. Comparison of the calculated and experimental unit cell volume and band gaps (*E*_g_) of these mixed-halide chalcohalides.

To better understand our experimental findings, we evaluated the crystal structure and electronic properties of the different mixed-halide compositions using DFT calculations. After first generating isocompositional coloring models^59,60^ to represent plausible atomic ordering patterns for Pb_3_SBrI_3_, Pb_3_SBr_2_I_2_, and Pb_3_SBr_3_I, we fully relaxed their structures using the Vienna *Ab initio* Simulation Package (VASP).^61^ We find that the lowest energy structures for the three different mixed-halides all adopt similar atomic coloring patterns. Specifically, the smaller bromine atoms are localized on the Pb(3) site^25^ toward the center of the unit cell in Pb_3_SBrI_3_ and Pb_3_SBr_2_I_2_, which then branch outward onto the Pb(2) site in Pb_3_SBr_3_I (Fig. 8). Interestingly, the lowest energy structure for Pb_3_SBrI_3_ corresponds to the *P*2_1_/*m* structure previously determined from single crystal data.^25^ Upon closer inspection of the relaxed structures, we observe a steady reduction of the unit cell volume from Pb_3_SBrI_3_ to Pb_3_SBr_2_I_2_ and Pb_3_SB_3_I (Fig. 8). Even though the calculated volumes are overestimated by ∼9–11% compared to volumes extracted from the refined XRD patterns (see ESI†), the overall trend is fully consistent with the increasing bromine incorporation into the chalcohalide structure.

A comparison of density of states (DOS) curves shows that the band gap (*E*_g_) values of the chalcohalides progressively widen from 1.92 eV in Pb_3_SBrI_3_ to 1.98 eV and 2.06 eV in Pb_3_SBr_2_I_2_ and Pb_3_SBr_3_I, respectively, which agrees well with the experimentally determined values (Fig. 8, see ESI†). The calculated band gaps for the three lowest energy structures underestimate their respective experimental values by *ca.* 11–13%, well within an acceptable range of underestimation often seen using VASP.^21,62^ Furthermore, analysis of the partial DOS reveals that the relative Br-p and I-p orbital contributions closely correlate with the relative halide ratio expected in each quaternary chalcohalide. In summary, the results of the electronic structure calculations closely support our experimentally observed trend of band gap widening with increased bromine incorporation into the mixed-halide chalcohalides.

## Conclusions

In conclusion, we have demonstrated an effective strategy for synthesizing quaternary chalcohalides with mixed-halide compositions using a solution-phase approach. Electron microscopy reveals that the Pb_3_SBr_*x*_I_4−*x*_ (1 ≤ *x* ≤ 3) semiconductors exhibit highly anisotropic growth in the form of rods and needles with high aspect ratios, and their structural features are validated using a combination of XRD and ^207^Pb ssNMR. Not only do these materials exhibit indirect band gaps that widen with increased Br incorporation, but they also show promising photoluminescence between 2.2–2.3 eV in solution as an ensemble as well as at the single particle level. These materials also demonstrate impressive stability upon exposure to moisture, showing minimal or no degradation when compared to traditional lead halide perovskites. Lastly, relative energy and DOS calculations showcase atomic coloring patterns and electronic structures that agree well with our experimental findings. Overall, this work provides fundamental materials chemistry knowledge that can be applied toward halide-alloying many other complex chalcohalide compositions, which remains a critical goal for tuning the optical properties of next generation semiconductors and devices.

## Data availability

All necessary information is included in the ESI.†

## Author contributions

ANR designed the experiments, synthesized, and characterized all materials, performed the electronic structure calculations, and wrote the manuscript. YC and AS performed ssNMR measurements and analyses. JOA performed single particle microscopy experiments. EG performed transmission electron microscopy. All authors contributed to the characterization, data analysis, and discussion. ANR and JV conceived the project, and JV, AJR, and EAS guided and supervised the work. All authors have given approval to the final version of the manuscript.

## Conflicts of interest

There are no conflicts to declare.

## Supplementary Material

SC-014-D3SC02733C-s001

SC-014-D3SC02733C-s002

SC-014-D3SC02733C-s003

SC-014-D3SC02733C-s004
