# Supplementary material for: Designing complex Pb3SBrxI4−x chalcohalides: tunable emission semiconductors through halide-mixing
Source: Chem Sci. 2023 Oct 16;14(43):12331–8. doi: 10.1039/d3sc02733c (PMC10631247; doi:10.1039/d3sc02733c)
Supplement: SC-014-D3SC02733C-s001 [file SC-014-D3SC02733C-s001.pdf]

Electronic Supplementary Material (ESI) for Chemical Science.
This journal is © The Royal Society of Chemistry 2023

Supporting information for:

Designing complex $Pb_3SBr_xI_{4-x}$ chalcohalides: tunable emission semiconductors through halide-mixing

Alison N. Roth,[ab] Yunhua Chen,[ab] Anuluxan Santhiran,[ab] Jemima Opare-Addo,[ab] Eunbyeol Gi,[ab] Emily A. Smith,[ab] Aaron J. Rossini,[ab] and Javier Vela*[ab]

[a]US DOE Ames National Laboratory, Ames, Iowa 50011, United States;
[b]Department of Chemistry, Iowa State University, Ames, Iowa 50011, United States.

*e-mail: vela@iastate.edu, phone: 515-294-5536

Experimental.

Materials. Oleylamine (oleylNH_2, technical grade, 70%), 1-octadecene (ODE, technical grade, 90%), lead(II) thiocyanate ($Pb(SCN)_2$, 99.5%) and lead(II) iodide (PbI_2, 99%), were purchased from Sigma Aldrich; lead(II) bromide ($PbBr_2$, 98%) from Acros Organics; oleic acid (OA, technical grade, 90%) from Alfa Aesar; hexanes (99.9%) and methanol (99.9%) from Fisher. All chemicals were used as received. *Caution: Oleylamine is a highly corrosive liquid that must be handled with extreme care and in small amounts whenever possible.*

Synthesis. All syntheses were performed in air under standard atmospheric conditions. Pb_3SBrI_3 was prepared by stirring $Pb(SCN)_2$ (0.2 mmol), PbI_2 (0.6 mmol) and $PbBr_2$ (0.2 mmol) in a mixture of ODE (10 mL, 31 mmol), oleic acid (0.25 mL, 0.8 mmol) and oleylamine (0.25 mL, 0.8 mmol) in a 100 mL round bottom flask at 110 °C for 5 min until the solids were completely dissolved or well dispersed in solution. The temperature was then raised to 180 °C and the reaction mixture remained at that temperature for 90 min before cooling to room temperature by removing the heating mantle. Various mixed-halide compositions were prepared in a similar manner by adjusting the relative concentrations and conditions to: 0.4 mmol PbI_2, 0.2 mmol $PbBr_2$, 180 °C, 60 min; 0.2 mmol PbI_2, 0.2 mmol $PbBr_2$, 180 °C, 60 min; or 0.2 mmol PbI_2, 0.4 mmol $PbBr_2$, 200 °C, 60 min. *Purification.* Crude solutions of the chalcohalides were first suspended in hexanes (5 mL) and methanol (5 mL), then centrifuged at 4500 rpm for 5 min. After discarding the supernatant, the pellet was resuspended in hexanes and methanol (5 mL of each) and centrifuged again to remove excess oleylamine and oleic acid ligands. This process was repeated until the remaining supernatant was colorless after centrifugation.

Structural Characterization. Powder X-ray diffraction (XRD) was measured on a Rigaku Ultima IV diffractometer (40 kV, 44 mA) using Cu Kα radiation on a zero-background quartz sample holder. Rietveld refinements of the XRD patterns were performed using the GSAS-II software package.[1] Scanning electron microscopy (SEM) images were acquired on a JEOL JSM-IT200 scanning electron microscope. Transmission electron microscopy (TEM) imaging was performed on a JEOL 2100 scanning transmission electron microscope. Samples were prepared by drop casting dilute solution in hexanes onto a carbon-coated 200 mesh copper grid.

Solid-State ^{207}Pb NMR Spectroscopy. A majority of solid-state NMR experiments were performed on a Bruker 14.1 T [$\nu_0(^1H)$ = 600 MHz] wide bore magnet equipped with a Bruker AVANCE NEO console and a 2.5 mm HX Magic Angle Spinning (MAS) probe. Solid-state NMR experiments on $Pb_3SBr_{1.2}I_{2.8}$ were performed on a Bruker wide-bore 9.4 T [$\nu_0(^1H)$ = 400 MHz] NMR spectrometer equipped with a Bruker Advance III HD console and a Bruker 2.5 mm broadband HX MAS probe. The probes were configured in ^1H-^{207}Pb mode. ^{207}Pb chemical shifts were referenced by using the published indirect referencing scale and that relates the ^{207}Pb reference frequency to the ^1H reference frequency (Larmor frequency ratio ^{207}Pb and ^1H is 20.920599).[S2] ^1H chemical shifts were referenced to neat tetramethylsilane (TMS) by using

adamantane [$\delta_{iso}(^1H)$ = 1.76 ppm] as a secondary standard. The ^{207}Pb spectra shown in the main text were obtained by using the variable offset cumulative spectra (VOCS) approach,[S3] where the transmitter offset was incremented across the spectral range in steps of 300 ppm (37.6 kHz) until no signal was observed. All of the sub-spectra were then co-added to form the total spectrum. Each ^{207}Pb NMR sub-spectrum was acquired using a spin echo pulse sequence, a MAS frequency of 25 kHz, and 67 kHz radiofrequency field ^{207}Pb pulses (3.74 μs $\pi/2$ and 7.48 μs π pulse durations), a recycle delay of 1 s, and 12800 scans. For each sample, recycle delays of 1 s, 2 s and 4 s were tested, and the 1 s recycle delay was found to provide the best sensitivity. Generally, fewer than 10 individual sub-spectra were acquired and co-added to form the total ^{207}Pb NMR spectrum of each sample. Peak fitting of ^{207}Pb Solid-state NMR was done using the solid line shape analysis module (SOLA) in topspin version 3.6.5.

Optical Characterization. Solution photoluminescence (PL) spectra were collected on a Horiba Jobin Yvon Fluorolog-3 spectrofluorometer (Slit width = 5 nm; λ_{exc}= 400 nm). The crude samples were suspended in hexanes for all solution PL measurements. Diffuse-reflectance spectra were collected using a SL1 Tungsten Halogen lamp (vis–IR), a SL3 Deuterium Lamp (UV), and a BLACK-Comet C-SR-100 spectrometer (200–1080 nm). The band gap values were estimated by extrapolating the linear slope of Tauc plots by plotting $(Ah\nu)^r$ versus $h\nu$ (A = absorbance, $h\nu$ = incident photon energy in eV, and r = 2 for indirect band gap semiconductors).[S4]

Single Particle Fluorescence Microscopy. Single particle photoluminescence (PL) was performed on an inverted microscope operated in epi-fluorescence mode (Nikon Eclipse TE2000U Melville, NY). Quaternary mixed-halides were diluted in hexanes and sonicated for 90 min before depositing 50 μL on a glass microscope coverslip (Fisher Scientific, Pittsburgh, PA). The solvent was removed under vacuum for 15 min. An Xcite Series 120 PC mercury lamp was used for excitation. A filter set from Omega Optical (Brattleboro, VT) was equipped with XF1009 (425DF45) excitation and XF3304 (605WB20) emission filter (λ_{exc} = 425±25 nm and λ_{em} = 605±15 nm). The dichroic filter used was XF2007 (475DRLP). A 100× Plan Apo, 1.49 numerical oil-immersion objective, was used for all experiments. Single particle PL images were collected on an Andor iXon Ultra EMCCD camera (Oxford Instruments, Abingdon, UK) with 40 ms exposure time and 100× electron multiplication (EM) gain. Each movie was 60 s in duration, and 5 movies were collected per sample. ImageJ was used to analyze the PL intensity versus time for five selected particles and backgrounds. The reported data represents an average intensity and background.

Calculations. Relative energy calculations were performed using the Vienna Ab initio Simulation Package (VASP).[S5] Electronic exchange-correlation was treated using the Perdew-Burke-Ernzerhof (PBE) functional.[S6] The cut-off energy for the plane wave basis functions was 500 eV and projected augmented-wave (PAW) pseudopotentials were used. During the structural optimizations the volume, atomic positions and cell shape were allowed to fully relax until the convergence energy was less than 1×10^{-4} eV. The relative total energies were calculated over a 16×16×16 k-point grid using the tetrahedron method. Density of states (DOS) were calculated after converging the total energy on a k-mesh of 20×20×20 in the irreducible wedge of the Brillouin

zone also using the tetrahedron method. Unit cell representations and atomic coloring patterns were generated using VESTA.[S7]

Table S1. Representative precursor concentrations used in the synthesis of mixed-halide chalcohalides.[a]

[PbI_2:$PbBr_2$]	*PbI_2 (mM)*	*$PbBr_2$ (mM)*	*$Pb(SCN)_2$ (mM)*	*%Br*
[3:1]	57	19	19	25
[2:1]	38	19	19	33
[1:1]	19	19	19	50
[1:2]	19	38	19	66

[a]All concentrations based on V_{tot} kept at 10.5 mL (ODE = 10 mL; oleylNH_2 = 0.25 mL; oleic acid = 0.25 mL).

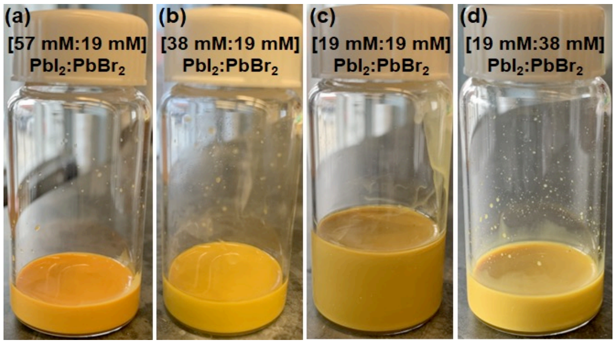

Figure S1. Crude solutions (prior to purification) of mixed-halide lead chalcohalides prepared using various concentrations of PbI_2 and $PbBr_2$.

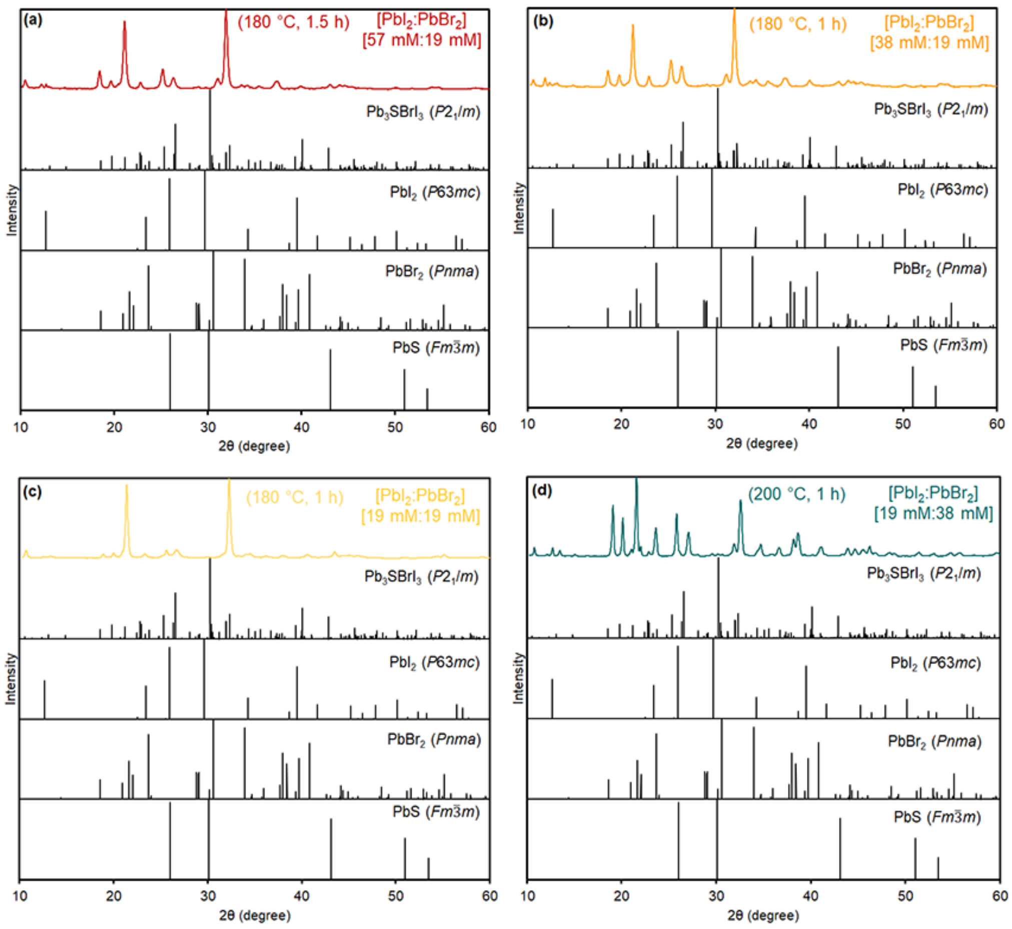

Figure S2. Powder XRD patterns of mixed-halide lead chalcohalides compared to those of common standards.

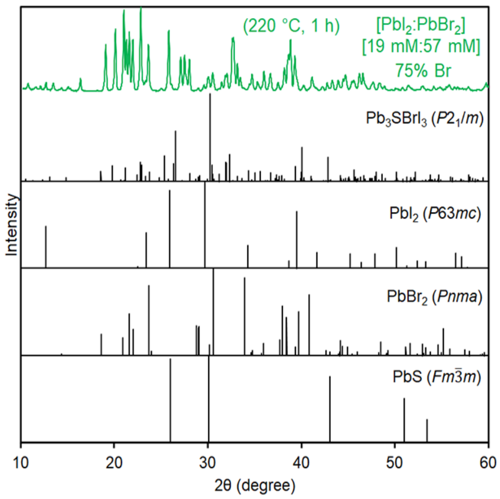

Figure S3. Powder XRD pattern obtained from a synthesis performed with 19 mM PbI_2 and 57 mM $PbBr_2$ (75% Br) at 220 °C for 1 h.

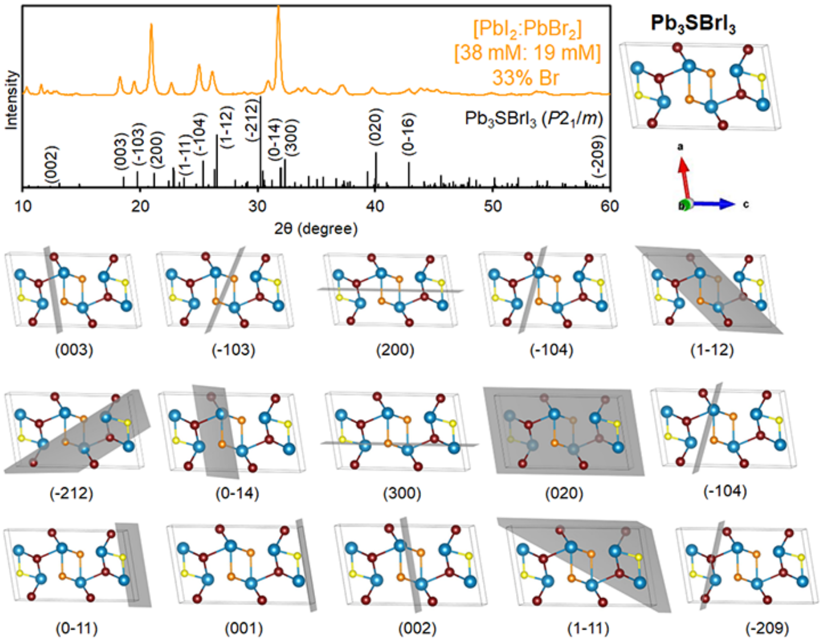

Figure S4. Powder XRD pattern obtained from a reaction of 38 mM PbI_2 and 19 mM $PbBr_2$ with lattice planes visualized for the Pb_3SBrI_3 ($P2_1/m$) unit cell.

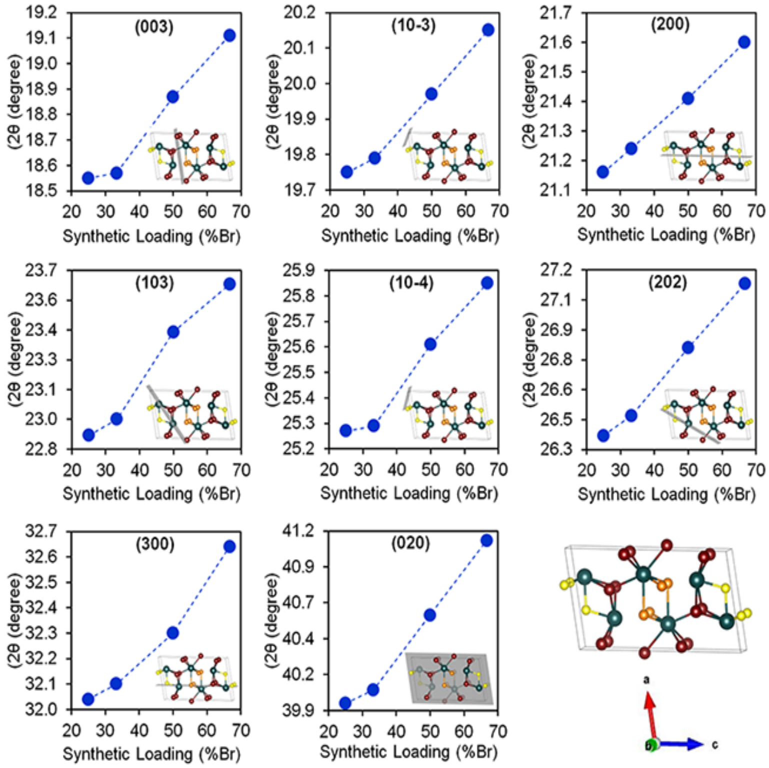

Figure S5. Peak shift of different XRD reflections as a function of relative halide synthetic loading (%Br).

Equation S1. Structure parameters and Miller indices in a monoclinic crystal system.

$$\frac{1}{d^2} = \frac{1}{sin^2\beta}\left(\frac{h^2}{a^2} + \frac{k^2sin^2\beta}{b^2} + \frac{l^2}{c^2} - \frac{2hlcos\beta}{ac}\right)$$

(hkl) = Miller indices

d = d-spacings (Å)

β = degree (°)

Table S2. Structure parameters of mixed-halide chalcohalides determined from raw XRD data and equation S1.

% Br	*a (Å)*	*b (Å)*	*c (Å)*	*Volume (Å^3)*
25	8.521	4.520	14.547	549.715
33.3	8.481	4.500	14.548	547.299
50	8.428	4.440	14.317	527.626
66.7	8.360	4.380	14.157	509.701

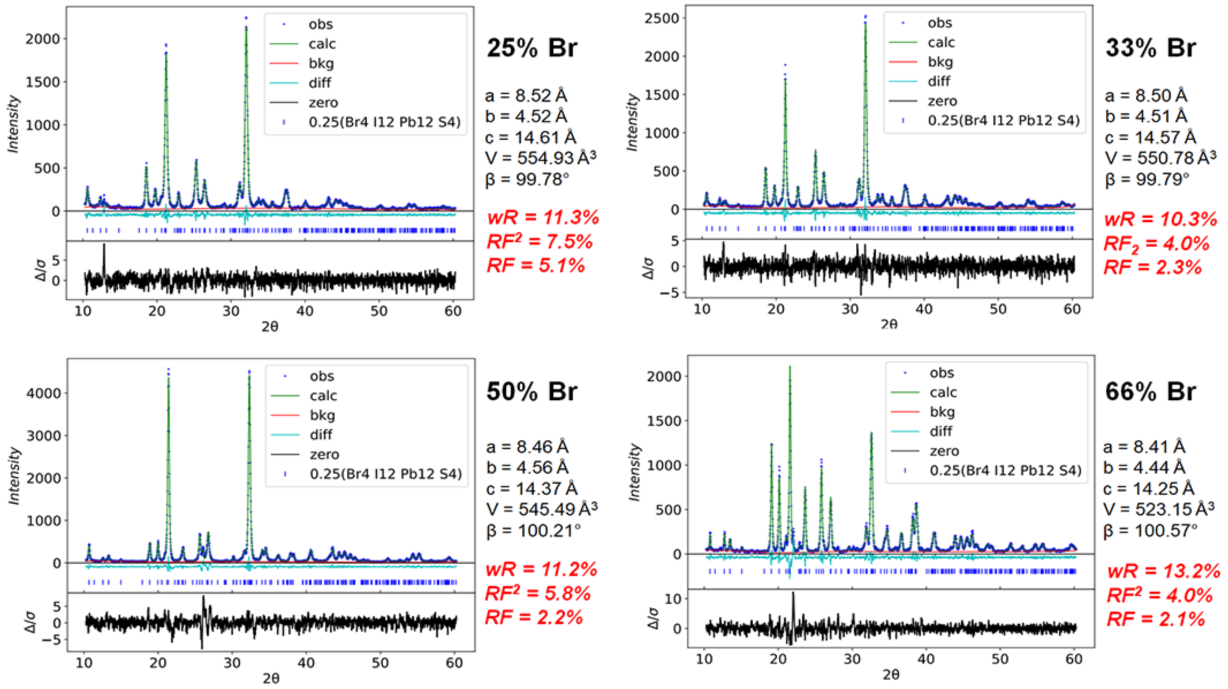

Figure S6. Rietveld refinement of mixed-halide chalcohalide XRD patterns with respect to the $P2_1/c$ Pb_3SBrI_3 standard pattern.

Table S3. Lattice parameters obtained from the Rietveld refinement of mixed-halide chalcohalide XRD patterns.

% Br	*a (Å)*	*b (Å)*	*c (Å)*	*Volume (Å^3)*	*β (°)*
25.0	8.52	4.52	14.61	554.93	99.78
33.3	8.50	4.51	14.57	550.78	99.79
50.0	8.46	4.56	14.37	545.49	100.21
66.7	8.41	4.44	14.25	523.15	100.57

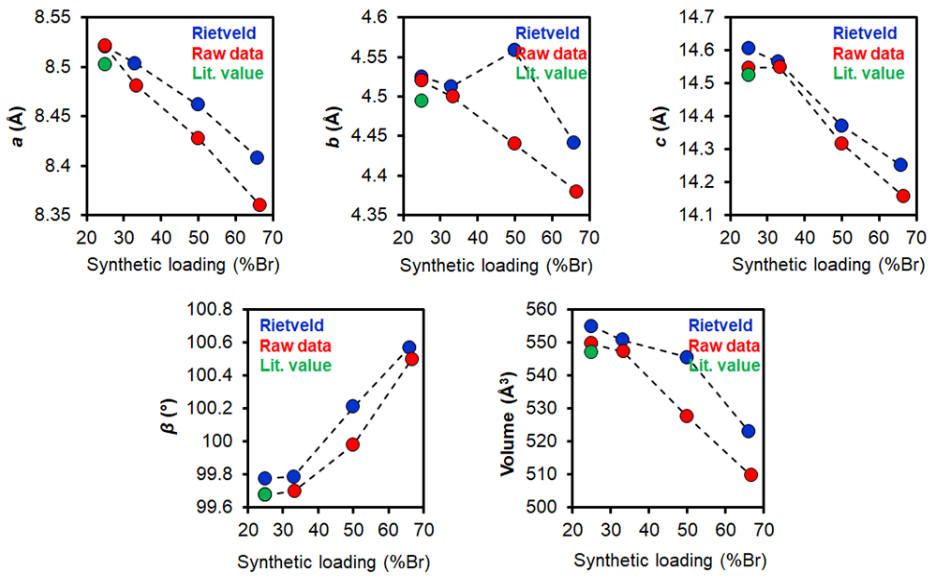

Figure S7. Comparison of lattice parameters determined from Equation 1 and Rietveld refinement and those reported in the literature[S8] as a function of relative halide synthetic loading (%Br).

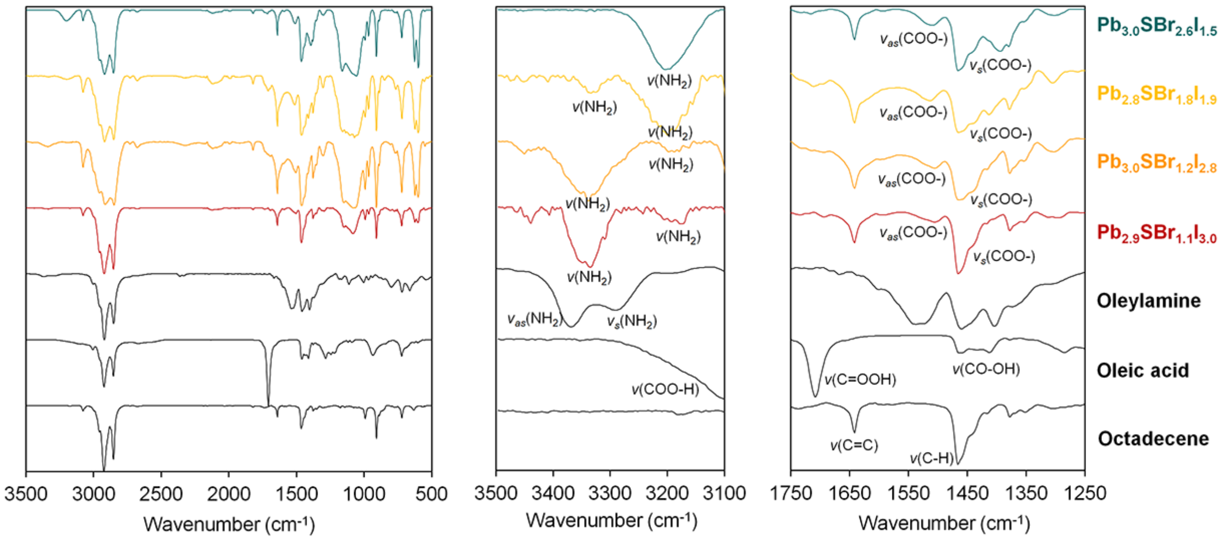

Figure S8. Full IR spectra (left), 3100-3500 cm^{-1} (amine) region (center), and 1250-1750 cm^{-1} (carboxylate) region (right) of mixed-halide chalcohalide semiconductors prepared from solution.

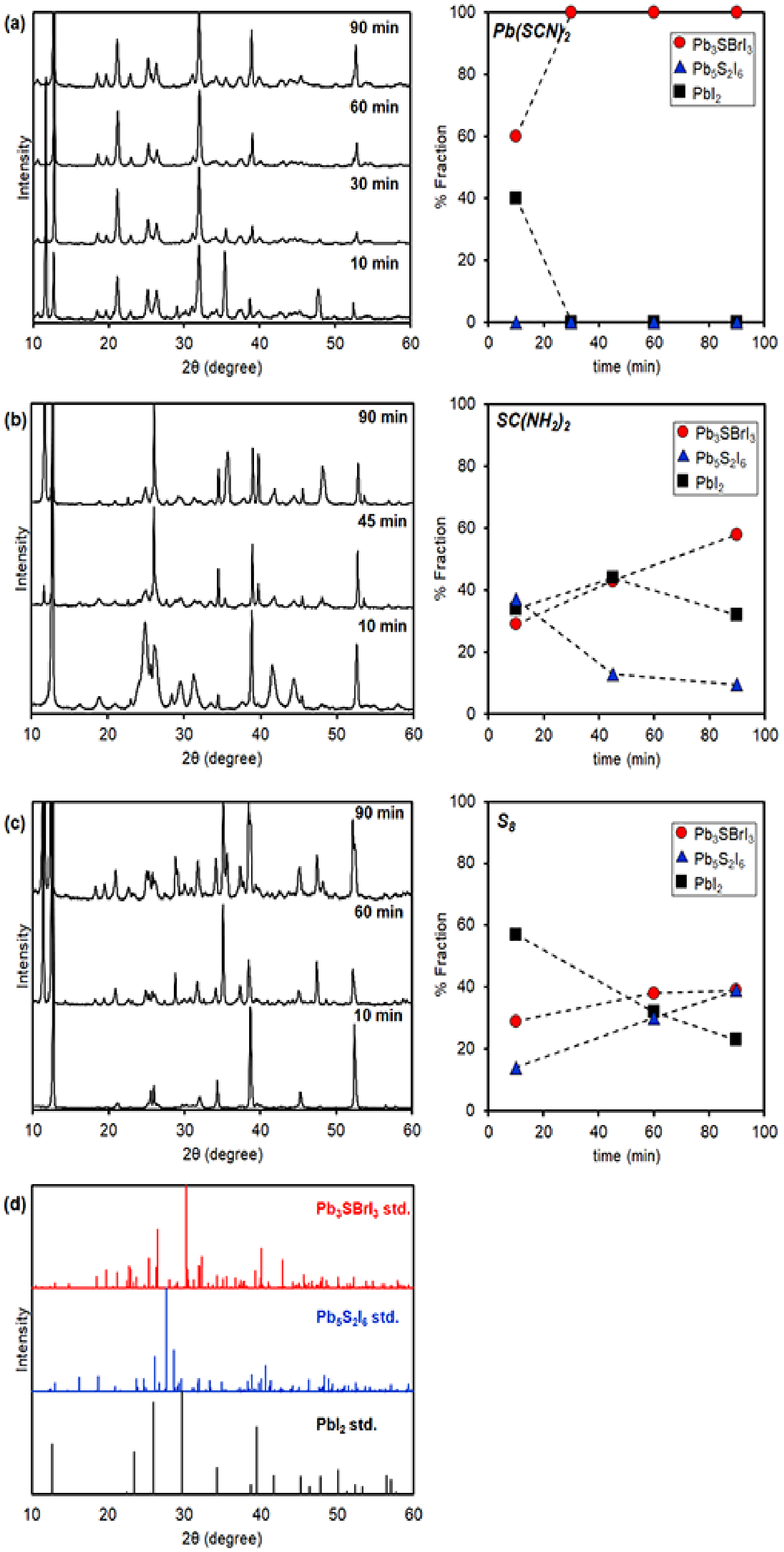

Figure S9. Synthesis and phase evolution of Pb_3SBrI_3 along with common impurity phases using different sulfur precursors: (a) Lead thiocyanate ($Pb(SCN)_2$), (b) thiourea ($SC(NH_2)_2$), and (c) elemental sulfur (S_8). (d) Stack plot of relevant quaternary, ternary, and binary standard patterns.

Table S4. Bond dissociation enthalpy of lead halides and chalcogenides.[S9]

Bond	ΔH_{diss} (kJ/mol)
Pb-I	194
Pb-Br	248
Pb-S	398
Pb-O	382

Table S5. Solubility product constants of binary lead compounds.[S9]

Compound	K_{sp}
PbI_2	9.8×10^{-9}
$PbBr_2$	6.6×10^{-6}
PbS	3.0×10^{-28}

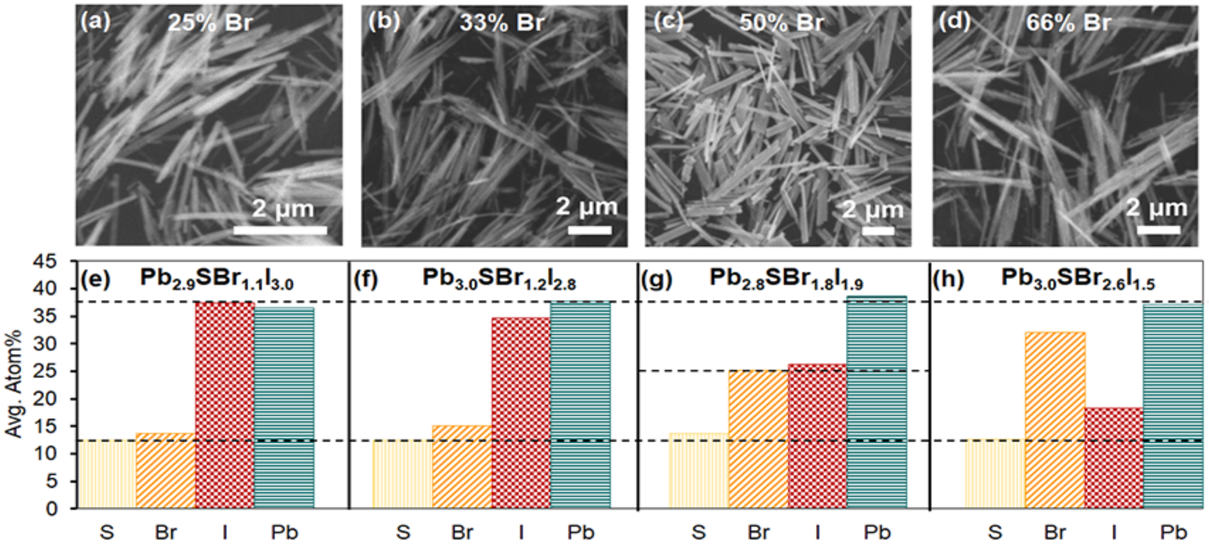

Figure S10. SEM images and EDS analysis of mixed-halide lead chalcohalides corresponding to (a, e) $Pb_{2.9}SBr_{1.1}I_{3.0}$, (b, f) $Pb_{3.0}SBr_{1.2}I_{2.8}$, (c, g) $Pb_{2.8}SBr_{1.8}I_{1.9}$, and (d, h) $Pb_{3.0}SBr_{2.6}I_{1.5}$, respectively. Dashed lines represent the theoretical atom% for each element in Pb_3SBrI_3, $Pb_3SBr_2I_2$ and Pb_3SBr_3I, respectively.

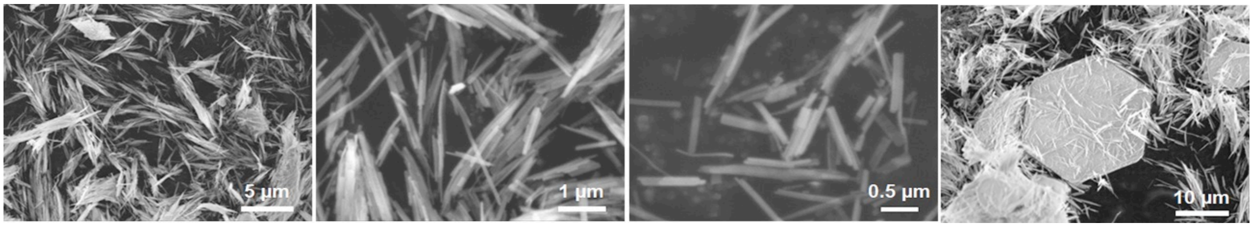

Figure S11. SEM images of $Pb_{2.9}SBr_{1.1}I_{3.0}$ obtained using 57 mM PbI_2 and 19 mM $PbBr_2$. A few hexagonal particles rich in lead and iodine were observed.

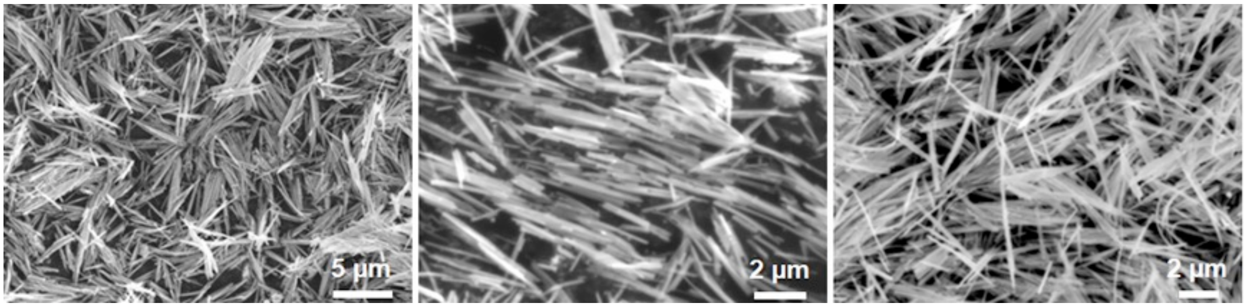

Figure S12. SEM images of $Pb_{3.0}SBr_{1.2}I_{2.8}$ obtained using 38 mM PbI_2 and 19 mM $PbBr_2$.

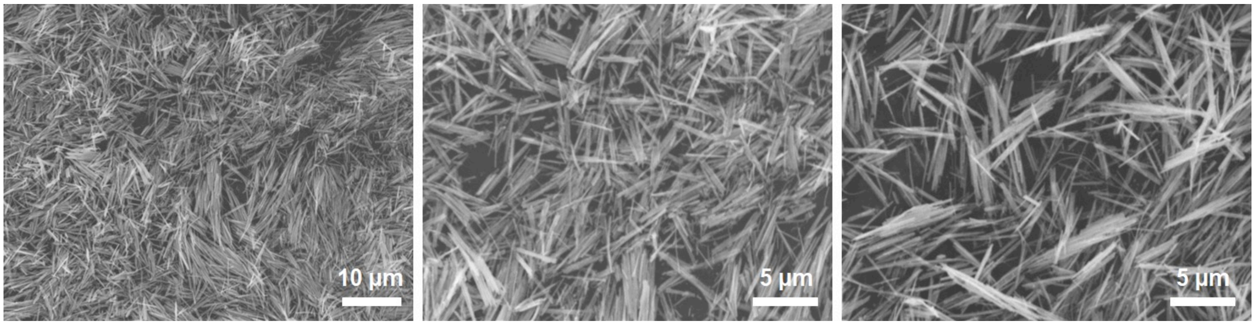

Figure S13. SEM images of $Pb_{2.8}SBr_{1.8}I_{1.9}$ obtained using 19 mM PbI_2 and 19 mM $PbBr_2$.

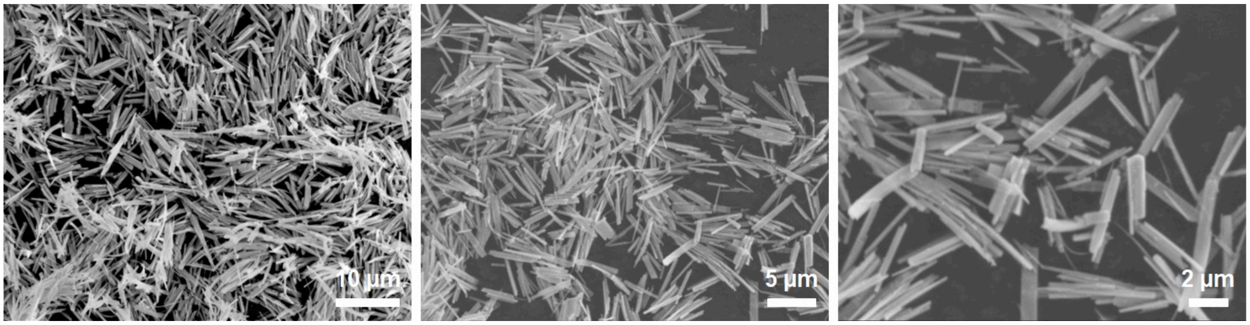

Figure S14. SEM images of $Pb_{3.0}SBr_{2.6}I_{1.5}$ obtained using 19 mM PbI_2 and 38 mM $PbBr_2$.

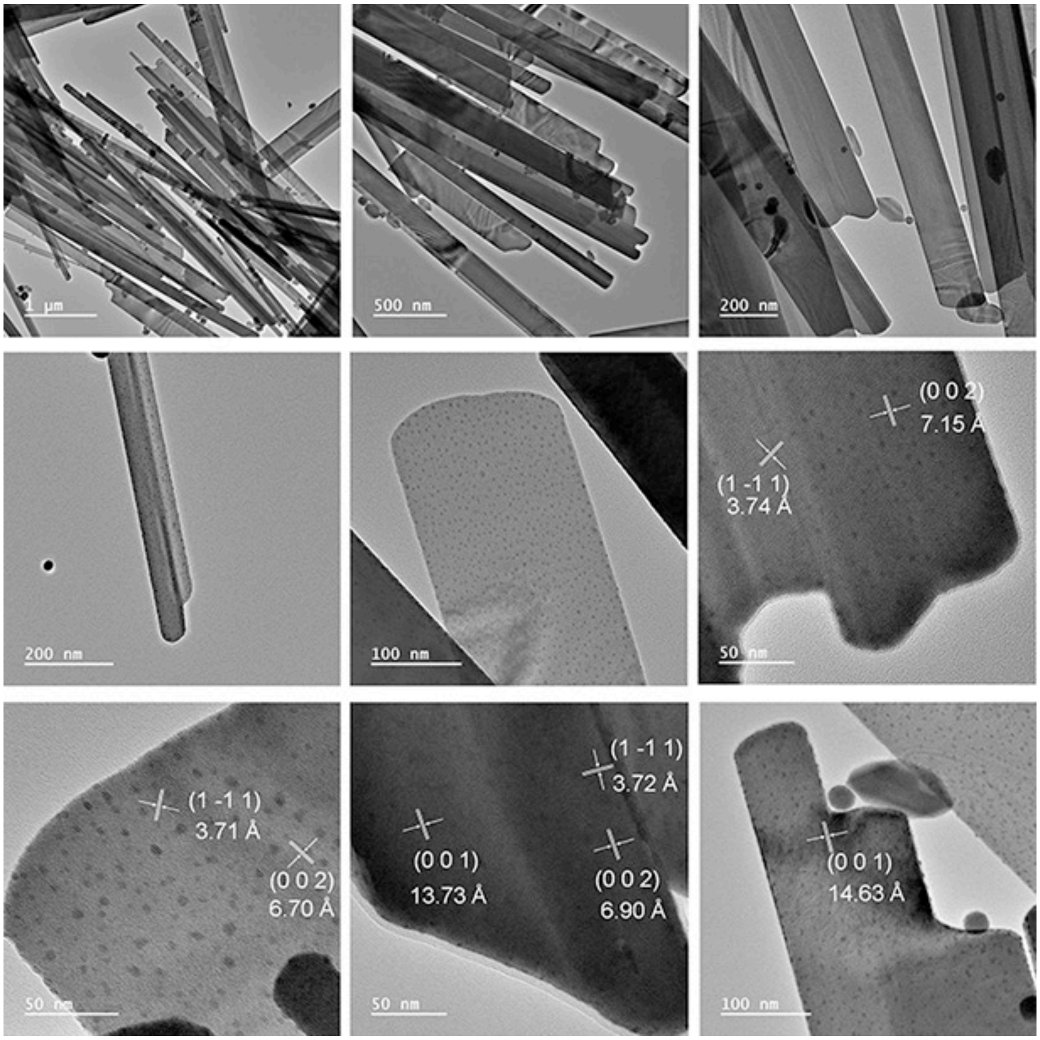

Figure S15. TEM and HRTEM images of $Pb_{2.8}SBr_{1.8}I_{1.9}$ obtained using 19 mM PbI_2 and 19 mM $PbBr_2$. A few small (< 4–5 nm) spots could be small amounts of lead-rich impurities such as PbI_2 or PbS particles; however, these phases are not observed in the powder XRD

Table S6. Experimental ^{207}Pb ssNMR chemical shift values for mixed-halide lead chalcohalides compared to reported values for potential binary lead impurity phases.

	σ_{iso} (ppm)	Reference
**$Pb_{3.0}SBr_{2.6}I_{1.5}$**		
Pb site 1	–	–
Pb site 2	−3.02	This work
Pb site 3	−691.1	This work
**$Pb_{2.8}SBr_{1.8}I_{1.9}$**		
Pb site 1	–	–
Pb site 2	−302.0	This work
Pb site 3	−639.6	This work
**$Pb_{3.0}SBr_{1.2}I_{2.8}$**		
Pb site 1	–	–

Pb site 2	0.366	This work
Pb site 3	-570.4	This work
**$Pb_{2.9}SBr_{1.1}I_{3.0}$**		
Pb site 1	701.6	This work
Pb site 2	−7.92	This work
Pb site 3	−531.7	This work
**PbI_2**	-31.1– -15.1	R. E. Taylor, P. A. Beckmann, S. Bai and C. Dybowski, *J. Phys. Chem. C,* 2014, **118**, 9143−9153.
**PbI_2**	-29.1	C. Dybowski, M. L. Smith, M. A. Hepp, E. J. Gaffney, G. Neue and D. L. Perry, *Appl. Spectrosc.,* 1998, **52**, 426−429.
**PbI_2**	-25	Y. S. Kye, S. Connolly, B. Herreros and G. S. Harbison, *Main Group Met. Chem.*, 1999, **22**. 373−383.
**PbI_2**	-29	A. Glatfelter, C. Dybowski, D. D. Kragten, S. Bai, D. L. Perry and J. Lockard, *Spectrochimica Acta Part A,* 2007, **66**, 1361–1363.
**$PbBr_2$**	-979	Y. S. Kye, S. Connolly, B. Herreros and G. S. Harbison, *Main Group Met. Chem.*, 1999, **22**. 373−383.
**$PbBr_2$**	-981	A. Glatfelter, C. Dybowski, D. D. Kragten, S. Bai, D. L. Perry and J. Lockard, *Spectrochimica Acta Part A,* 2007, **66**, 1361–1363.
**α-PbO**	1949	Y. S. Kye, S. Connolly, B. Herreros and G. S. Harbison, *Main Group Met. Chem.*, 1999, **22**. 373−383.
**α-PbO**	1939	F. Fayon, I. Farnan, C. Bessada, J. Coutures, D. Massiot and J. P. Coutures, *J. Am. Chem. Soc.,* 1997, **119**, 6837-6843.
**α-PbO**	1930	S. P. Gabuda, S. G. Kozlova, V. V. Terskikh, C. Dybowski, G. Neue and D. L. Perry, *Chem. Phys. Lett.,* 1999, **305**, 353−358.
**β-PbO**	1502	Y. S. Kye, S. Connolly, B. Herreros and G. S. Harbison, *Main Group Met. Chem.*, 1999, **22**. 373−383.
**β-PbO**	1515	F. Fayon, I. Farnan, C. Bessada, J. Coutures, D. Massiot and J. P. Coutures, *J. Am. Chem. Soc.,* 1997, **119**, 6837-6843.
**β-PbO**	1527	S. P. Gabuda, S. G. Kozlova, V. V. Terskikh, C. Dybowski, G. Neue and D. L. Perry, *Chem. Phys. Lett.,* 1999, **305**, 353−358.
**PbS**	1380	M. Jagadeeswararao, P. Vashishtha, T. J. N. Hooper, A. Kanwat, J. W. M. Lim, S. K. Vishwanath, N. Yantara, T. Park, T. C. Sum, D. S. Chung, S. G. Mhaisalkar and N. Mathews, *J. Phys. Chem. Lett.,* 2021, **12**, 9569−9578.
**PbS**	ca.1400–1700	M. Sytnyk, S. Yakunin, W. Schöfberger, R. T. Lechner, M. Burian, L. Ludescher, N. A. Killilea, A. YousefiAmin, D. Kriegner, J. Stangl, H. Groiss and W. Heiss, *ACS Nano,* 2017, **11**, 1246−1256.

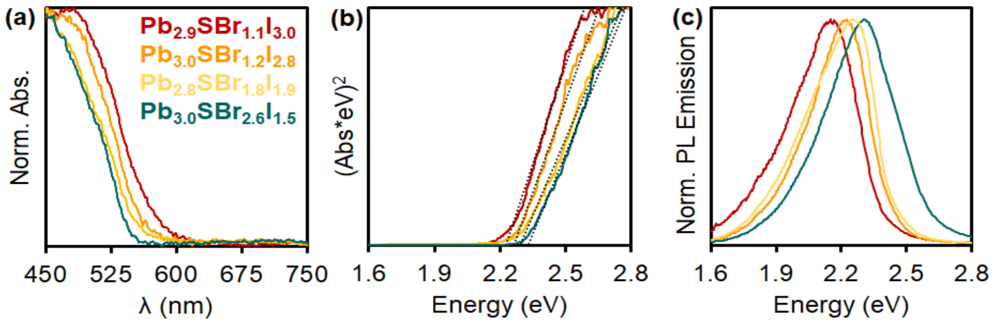

Figure S16. (a) Diffuse reflectance, (b) Tauc plot, and (c) photoluminescence spectra of mixed-halide chalcohalides (λ_{exc} = 400 nm).

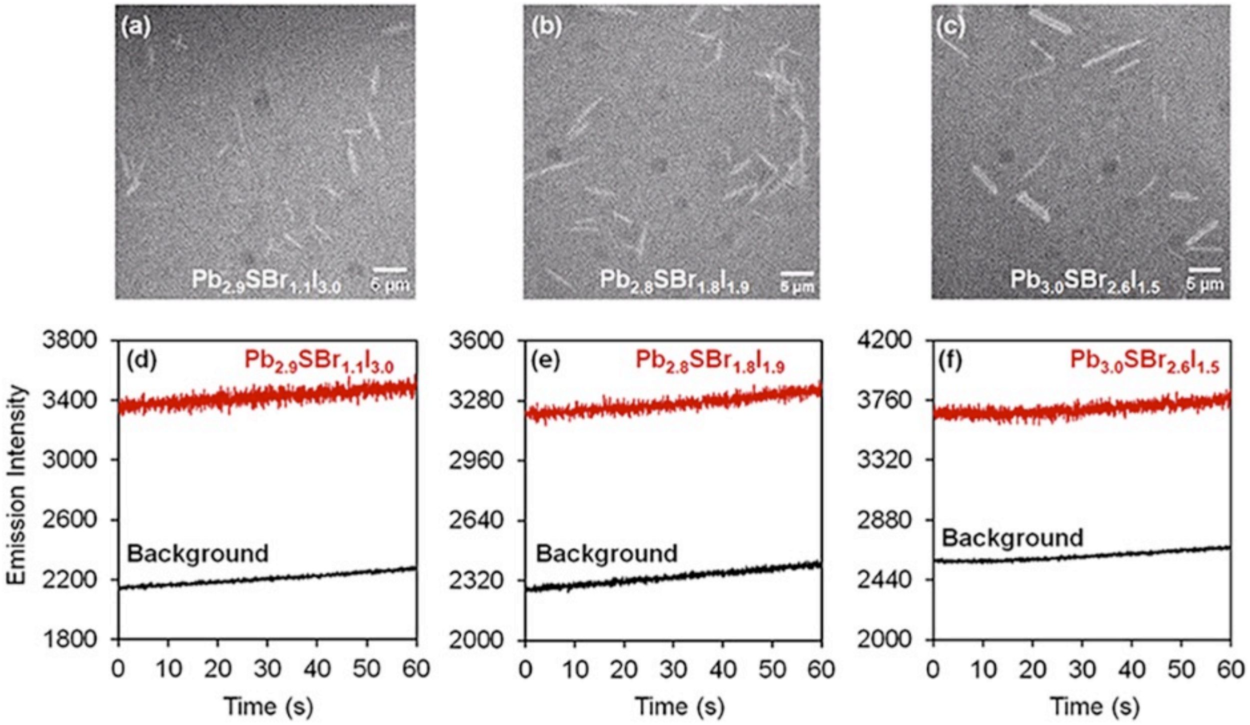

Figure 17. Single particle photoluminescence (PL) images (top) and PL emission intensity collected over time under continuous illumination (bottom) of (a, d) $Pb_{2.9}SBr_{1.1}I_{3.0}$, (b, e) $Pb_{2.8}SBr_{1.8}I_{1.9}$, and (c, f) $Pb_{3.0}SBr_{2.6}I_{1.5}$.

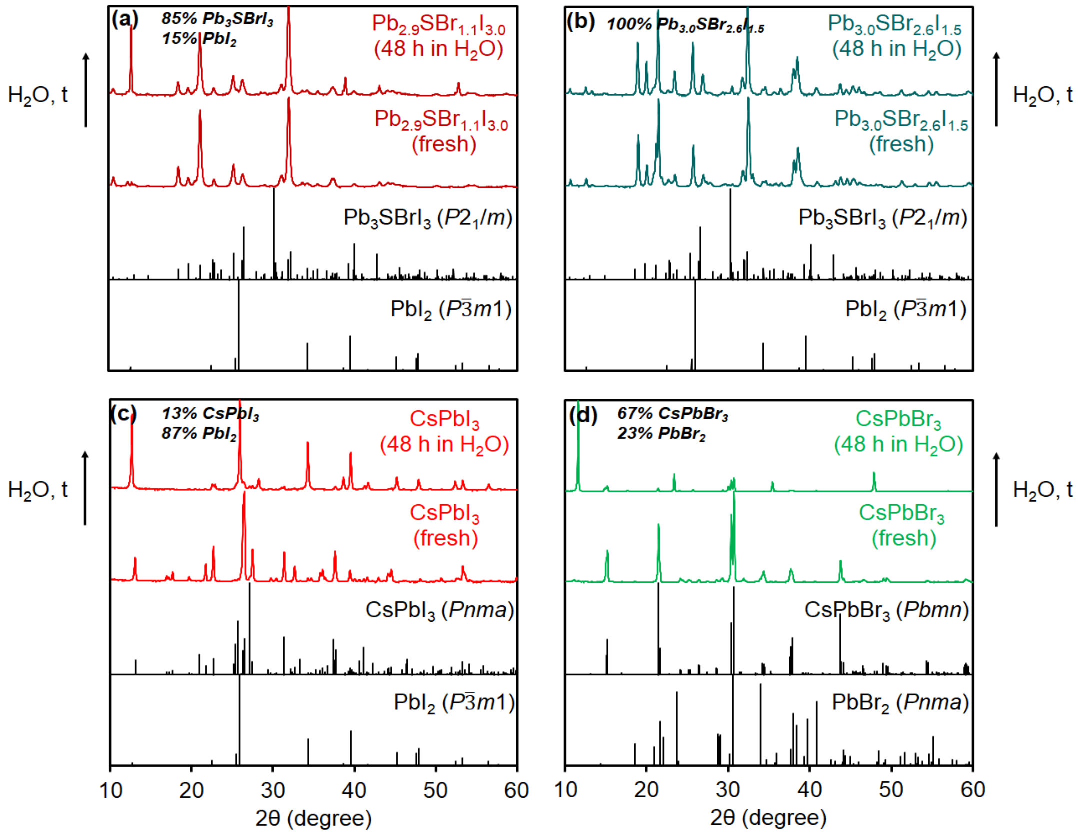

Figure S18. Stability of mixed-halide chalcohalides vs. mixed-halide perovskites in water over time.

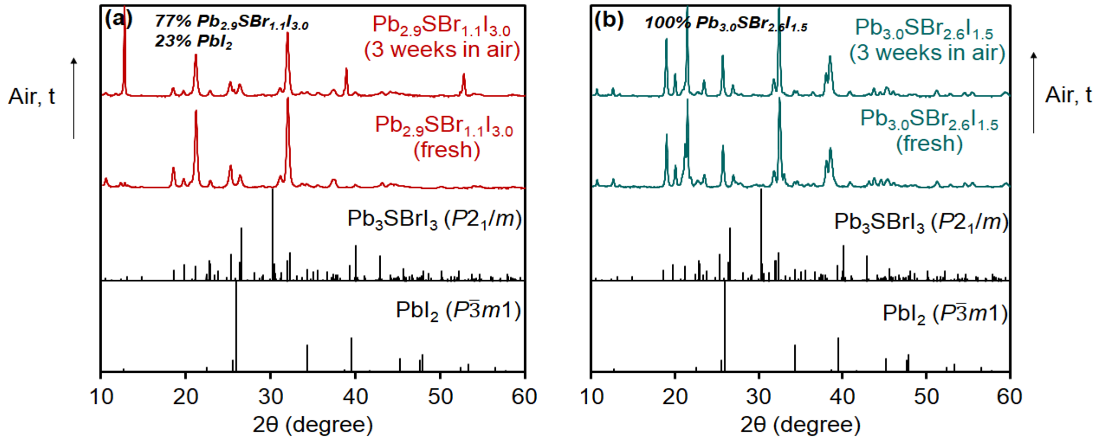

Figure S19. Stability of $Pb_{2.9}SBr_{1.1}I_{3.0}$ and $Pb_{3.0}SBr_{2.6}I_{1.5}$ under air room temperature over time.

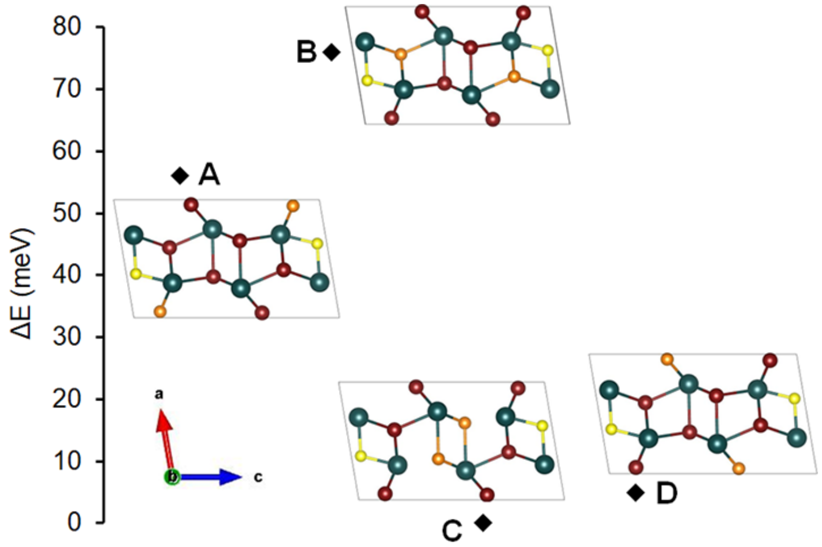

Figure S20. Calculated relative energies of Pb_3SBrI_3 with different atomic coloring patterns.

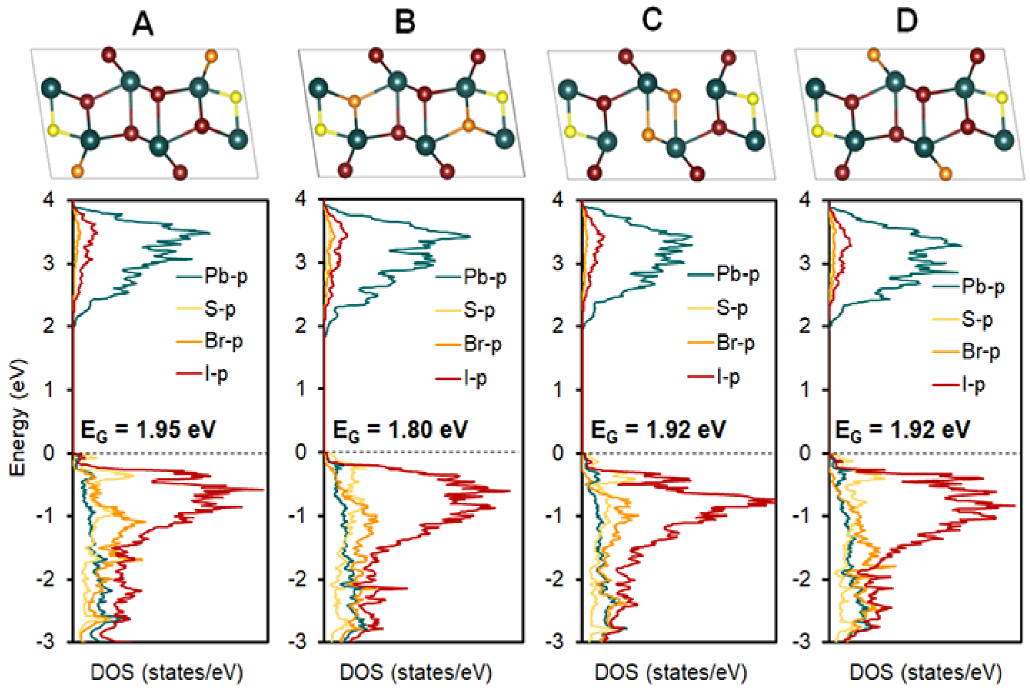

Figure S21. Calculated pDOS of Pb_3SBrI_3 with different atomic coloring patterns.

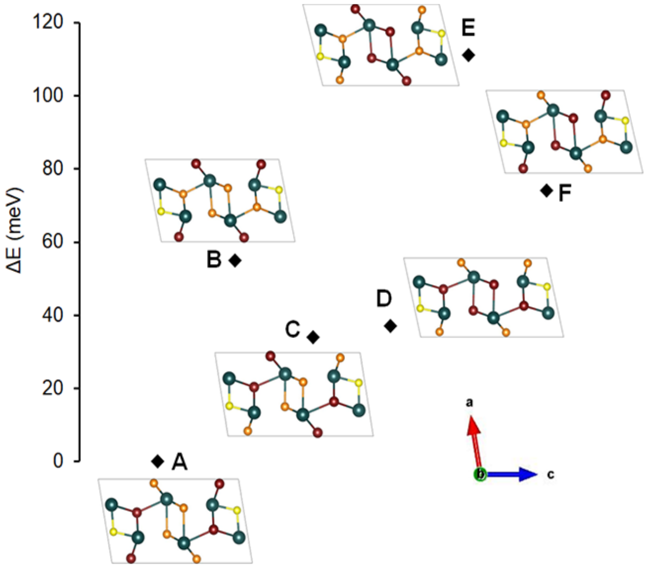

Figure S22. Calculated relative energies of $Pb_3SBr_2I_2$ with different atomic coloring patterns.

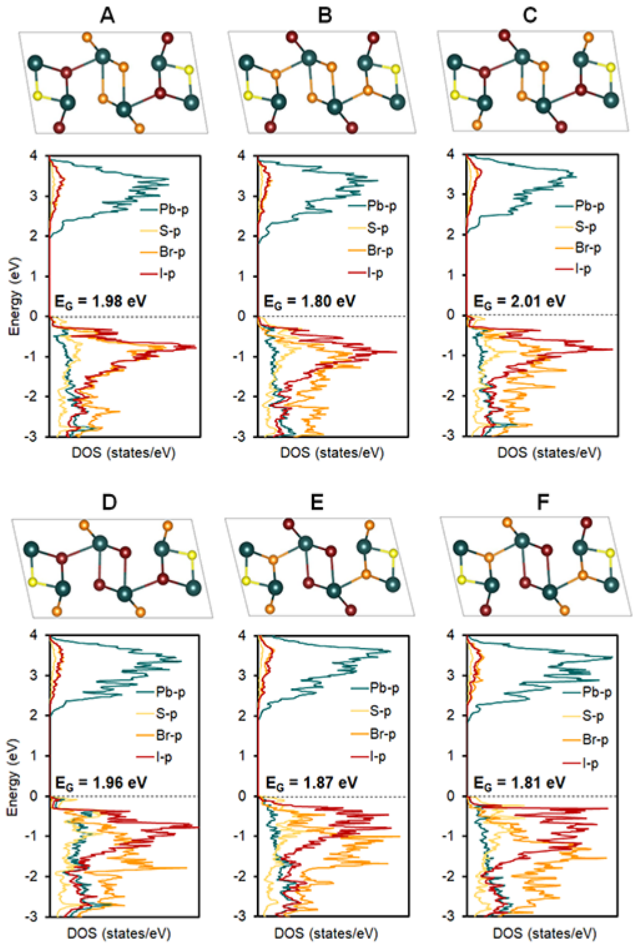

Figure S23. Calculated pDOS of $Pb_3SBr_2I_2$ with different atomic coloring patterns.

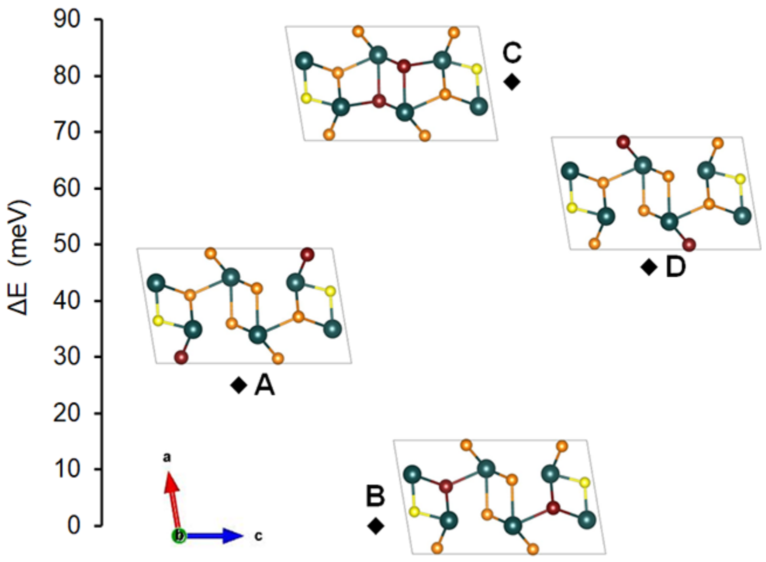

Figure S24. Calculated relative energies of Pb_3SBr_3I with different atomic coloring patterns.

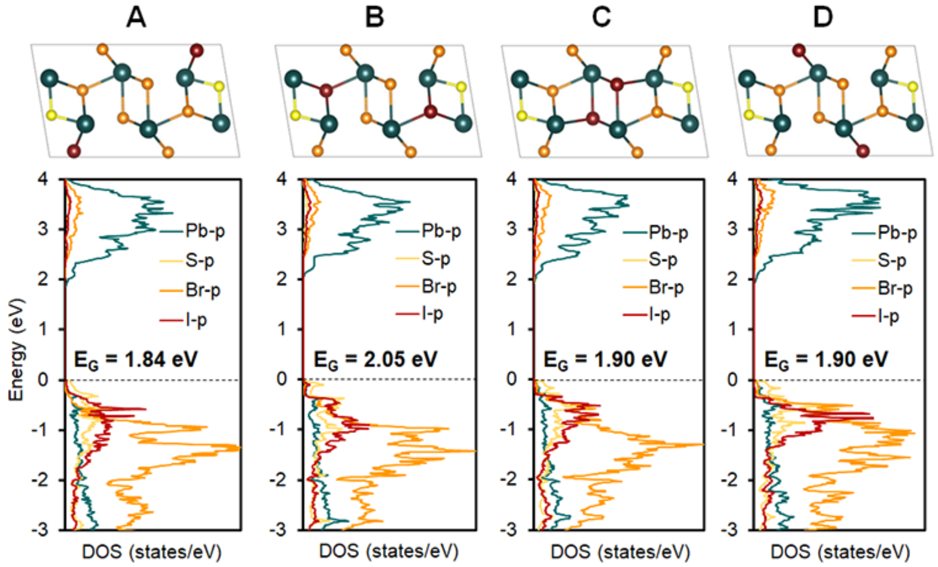

Figure S25. Calculated pDOS of Pb_3SBr_3I with different atomic coloring patterns.

Table S7. Lattice parameters of the fully relaxed, lowest energy structures of mixed-halide chalcohalides (obtained using VASP[S5,S6]) (see Figures S20, S22, and S24).

	a (Å)	*b* (Å)	*c* (Å)	Volume (Å^3)	*β* (°)
Pb_3SBrI_3 (“**C**”)	8.70	4.56	15.23	597.32	99.08
$Pb_3SBr_2I_2$ (“**A**”)	8.64	4.51	15.19	583.37	99.99
Pb_3SBr_3I (“**B**”)	8.37	4.45	15.42	564.54	100.50

Table S8. Difference between relaxed unit cell volume and experimental unit cell volume of mixed-halide chalcohalides (see Figures S20, S22, and S24).

	Pb_3SBrI_3 ("**C**")	$Pb_3SBr_2I_2$ ("**A**")	Pb_3SBr_3I ("**B**")
Relaxed unit cell volume (Å^3)	597.32	583.37	564.54
Experimental unit cell volume (Å^3)	549.72	527.63	509.70
%Δ	8.7	10.6	10.8

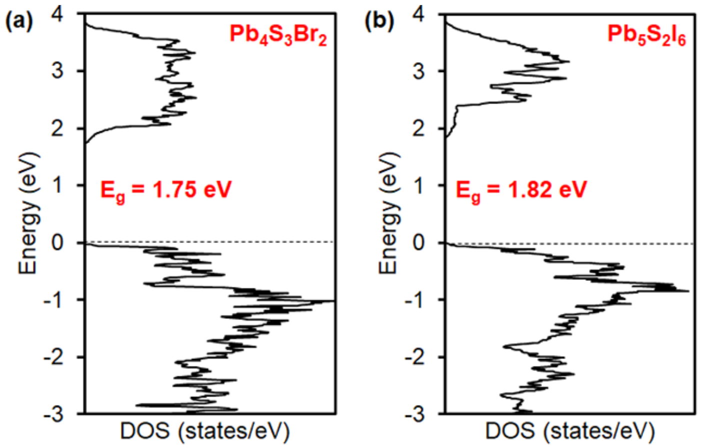

Figure S26. Total DOS of (a) $Pb_4S_3Br_2$ and (b) $Pb_5S_2I_6$ calculated using VASP[S5,S6] (both structures were first allowed to fully relax to their lowest energy structures).

Table S9. Comparison of calculated and experimental band gap (E_g) values of quaternary and ternary lead chalcohalides (see Figures S21, S23, S25, and S26).

	Calculated E_g (eV)^a	Experimental E_g (eV)	Underestimation of E_g (%)
Pb_3SBrI_3 ("**C**")	1.92	2.22	13.5
$Pb_3SBr_2I_2$ ("**A**")	1.98	2.29	13.5
Pb_3SBr_3I ("**B**")	2.06	2.33	11.6
$Pb_4S_3Br_2$	1.75	1.91^b	8.4
$Pb_5S_2I_6$	1.82	1.98^c	8.1

^{a}Obtained from VASP;[S5,S6] ^{b}literature value;[S10] ^{c}literature value.[S11]

[S1] B. H. Toby and R. B. Von Dreele, *J. Appl. Crystallogr.*, 2013, **46**, 544–549.

[S2] R. K. Harris, E. D. Becker, S. M. Cabral de Menezes, R. Goodfellow and P. Granger, *Pure Appl. Chem.,* 2001, **73**, 1795–1818.

[S3] B. Bureau, G. Silly, J. Y. Buzaré, C. Legein and D. Massiot, *Solid State Nucl. Magn. Reason.,* 1999, **14**, 181–190.

[S4] B. D. Viezbicke, S. Patel, B. E. Davis and D. P. Birnie, *Phys. Status Solidi B,* 2015, **252**, 1700−1710.

[S5] G. Kresse and J. Hafner, *Phys. Rev. B: Condens. Matter Mater. Phys*., 1994, **49**, 14251–14269.

[S6] J. P. Perdew, K. Burke and M. Ernzerhof, *Phys. Rev. Lett.,* 1996, **77**, 3865– 3868.

[S7] K. Momma and F. J. Izumi, *J. Appl. Crystallogr.,* 2011, **44**, 1272–1276.

[S8] M. Yan, R. L. Tang, W. Zhou, W. Liu and S. P. Guo, *Dalton Trans.,* 2022, **51**, 12921−12927.

[S9] W. M. Haynes, D. R. Lide and T. J. Bruno, *CRC Handbook of Chemistry and Physics, 103rd edition,* CRC Press, Boca Raton, FL, 2022.

[S10] S. Toso, Q. A. Akkerman, B. Martín-García, M. Prato, J. Zito, I. Infante, Z. Dang, A. Moliterni, C. Giannini, E. Bladt, I. Lobato, J. Ramade, S. Bals, J. Buha, D. Spirito, E. Mugnaioli, M. Gemmi and L. Manna, *J. Am. Chem. Soc*., 2020, **142**, 10198–10211.

[S11] L. Sun, C. Wang, L. Xu, J. Wang, X. Chen and G. Yi, *J. Mater. Chem. C*, 2018, **6**, 7188–7194.
